# Greedy feature selection for glycan chromatography data with the generalized Dirichlet distribution

**DOI:** 10.1186/1471-2105-14-155

**Published:** 2013-05-07

**Authors:** Marie C Galligan, Radka Saldova, Matthew P Campbell, Pauline M Rudd, Thomas B Murphy

**Affiliations:** 1 School of Mathematical Sciences, University College Dublin, Belfield, Dublin 4, Ireland; 2 NIBRT Dublin Oxford Glycobiology Laboratory, NIBRT, Mount Merrion, Blackrock, Dublin 4, Ireland; 3 Department of Chemistry and Biomolecular Sciences, Biomolecular Frontiers Research Centre, Macquarie University, Sydney, New South Wales 2109, Australia

**Keywords:** Compositional data, Beta distribution, Generalized Dirichlet distribution, Variable selection, Feature selection, Correlation-based feature selection, Recursive partitioning, Glycobiology, Glycan, HILIC, Chromatography data

## Abstract

**Background:**

Glycoproteins are involved in a diverse range of biochemical and biological processes. Changes in protein glycosylation are believed to occur in many diseases, particularly during cancer initiation and progression. The identification of biomarkers for human disease states is becoming increasingly important, as early detection is key to improving survival and recovery rates. To this end, the serum glycome has been proposed as a potential source of biomarkers for different types of cancers.

High-throughput hydrophilic interaction liquid chromatography (HILIC) technology for glycan analysis allows for the detailed quantification of the glycan content in human serum. However, the experimental data from this analysis is compositional by nature. Compositional data are subject to a constant-sum constraint, which restricts the sample space to a simplex. Statistical analysis of glycan chromatography datasets should account for their unusual mathematical properties.

As the volume of glycan HILIC data being produced increases, there is a considerable need for a framework to support appropriate statistical analysis. Proposed here is a methodology for feature selection in compositional data. The principal objective is to provide a template for the analysis of glycan chromatography data that may be used to identify potential glycan biomarkers.

**Results:**

A greedy search algorithm, based on the generalized Dirichlet distribution, is carried out over the feature space to search for the set of “grouping variables” that best discriminate between known group structures in the data, modelling the compositional variables using beta distributions. The algorithm is applied to two glycan chromatography datasets. Statistical classification methods are used to test the ability of the selected features to differentiate between known groups in the data. Two well-known methods are used for comparison: correlation-based feature selection (CFS) and recursive partitioning (rpart). CFS is a feature selection method, while recursive partitioning is a learning tree algorithm that has been used for feature selection in the past.

**Conclusions:**

The proposed feature selection method performs well for both glycan chromatography datasets. It is computationally slower, but results in a lower misclassification rate and a higher sensitivity rate than both correlation-based feature selection and the classification tree method.

## Background

In the statistical literature, a *composition* is a vector of non-negative elements that are constrained to sum to a constant. *Compositional data* are composed of such vectors. They represent parts of a whole and are typically expressed as proportions or percentages. The variables in a composition are often referred to as *components*. Compositional data arise naturally in many disciplines, such as in plant ecology [1], archaeometry [2], and geology [3]. Notwithstanding this fact, is not uncommon for statistical analysis to be carried out without regard to the compositional nature of the data. The constant-sum constraint on the data restricts the sample space to a simplex and also induces spurious correlation between components [4], with the result that traditional statistical methods such as multivariate analysis of variance (MANOVA), pairwise correlations, and discriminant analysis are not directly suitable for these data.

Aitchison [3] provides great insight into the special considerations required in compositional data analysis, advocating the use of a *log-ratio* approach. This has met with much success, in the statistical and geological communitites in particular. Others have since built on his work, making available a collection of methods that are easily accessible for compositional data analysis.

We propose a feature selection method for compositional data. Notably little research appears to have been conducted into feature selection for compositions to date. This methodology was developed with a specific application in mind; feature selection for hydrophilic interaction liquid chromatography (HILIC) data from glycan analysis.

Glycans are complex sugar chains that are present in all cells. They can exist either in free form or are covalently bound to other macromolecules, such as proteins or lipids [5]. The diversity and complexity of these structures means that they have a broad range of functions, playing a structural role as well as being involved in most physiological processes [5]. Glycosylation is important in the growth and development of a cell, tumour growth and metastasis, immune recognition and response, anticoagulation, communication between cells, and microbial pathogenesis [6]. Glycans are generally attached to proteins through a nitrogen atom (*N*-glycans) or an oxygen atom (*O*-glycans).

Glycobiology has great potential for biomarker discovery, as it has been relatively unexploited in comparison with genomics and proteomics [7]. Alterations in the glycosylation profiles of proteins have been observed during the pathogenesis of many different diseases; including cancer, congenital disorders of glycosylation and inflammatory conditions such as rheumatoid arthritis and schizophrenia [8].

**Figure 1:**
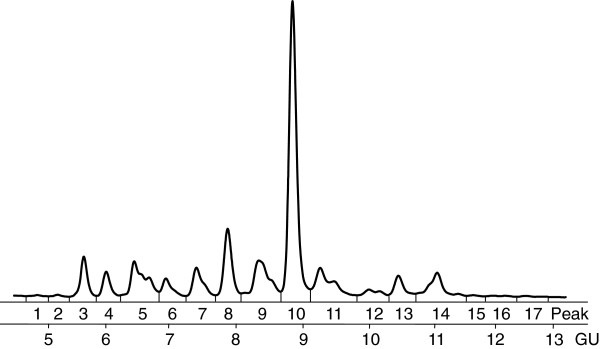
**Lung cancer HILIC profile.** Typical HILIC chromatogram of *N*-glycans released from serum glycoproteins for the Lung Cancer Cohort (1hr. HILIC, integrated into 17 peaks). Each peak represents one or more *N*-glycan structures.

**Figure 2:**
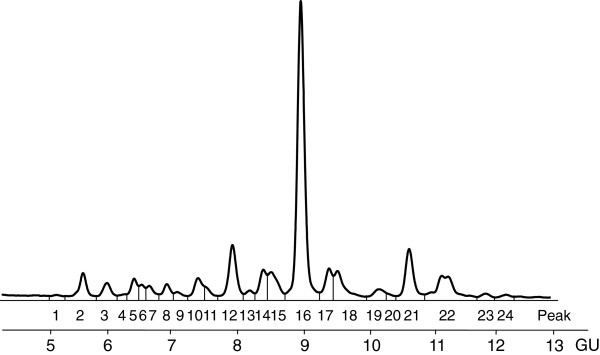
**Prostate cancer HILIC profile.** Typical HILIC chromatogram of *N*-glycans released from serum glycoproteins from the Prostate Cancer study (1hr. HILIC, integrated into 24 peaks). Each peak represents one or more *N*-glycan structures.

Developing analytical methods for the structural characterizations of glycans has proved to be challenging, due to their complex and heterogeneous nature. Royle et al. [9] recently developed a high-throughput *N*-glycan hydrophilic interaction liquid chromatography (HILIC) platform and described the detailed quantitative analysis of *N*-glycan structures from human serum (containing 117 glycans). HILIC analysis has emerged as one of the dominant analytical techniques for glycan analysis [10]. Chromatographic analysis produces a glycan profile or chromatogram, such as those in Figures 1 and 2 (after standardization using a dextran ladder). The relative area under each chromatographic peak represents the proportion of a particular subgroup of glycan structures present in the sample. The data are compositional, since each observation consists of the set of relative peak areas from an individual’s glycan profile. Often, the objective of conducting glycan analysis is to identify chromatographic peaks that differ between a set of known groups (e.g. control vs. disease).

A feature selection methodology for these data would provide a useful tool for biomarker research. One reason for this is that it could reduce the time and cost associated with further analysis. To identify the exact glycan structures corresponding to each chromatographic peak, further experimental analysis is required. To reduce the expense incurred from the addition of costly enzymes to the sample and the time required for detailed quantitative analysis, it would be extremely beneficial to be able to select a smaller subset of seemingly informative peaks for further refinement.

The level of refinement of the profile (the number of chromatographic peaks) is dependent on experimental conditions. The datasets demonstrated in this paper are from profiles consisting of 17 (lung cancer data) and 24 (prostate cancer data) chromatographic peaks. The dimensionality of glycan chromatography datasets is expected to increase in the future, as more advanced techniques have already become available [10]. For the purposes of biomarker discovery, it will become more important to have a methodology available for selecting subsets of chromatographic peaks that differ between control/disease groups.

Galligan et al [11] compared three suitable models for the classification of glycan chromatography data and found that modelling the data using the log-ratio approach of Aitchison [3] gave satisfactory results. A disadvantage is that fitting this model to compositional data requires transformation of the data, making interpretation of the model difficult in terms of the raw data (the glycan peaks). Proposed here is a feature selection methodology based on Connor and Mosimann’s generalized Dirichlet distribution [12] and its marginal, the beta distribution. This is an extension of the Dirichlet distribution that has almost double the number of parameters and allows for more flexible modelling of compositional data. The Dirichlet class is a natural parametric family for modelling data in a simplex sample space and therefore, no data transformation is required. There has been much interest in this class of models, and several other extensions of the ordinary Dirichlet distribution have been explored, such as the hyper Dirichlet [13] and the nested Dirichlet [14] distributions. The Dirichlet class has also been used for fitting regression models [15,16] and time series models to compositional data [17]. In addition, Wang et al. [18] proposed a dimension reduction technique for compositional data, using properties of the Dirichlet distribution. They project compositional data onto a lower dimensional simplex space, finding an “optimal” projection, defined as that which maximizes the estimated Dirichlet precision on the reduced data. A major advantage of modelling compositional data using the Dirichlet distribution is that transformation of the data is not required, hence, the results are directly interpretable in terms of the original variables. This is a desirable property for feature selection, as features can be directly selected from the model.

Raftery and Dean [19] propose a methodology for variable selection integrated with model-based clustering. They use the headlong search strategy proposed by Badsberg [20] to search over the feature space. They add and remove features during the model-building process using a comparison of the Bayesian Information Criterion (BIC) [21] for proposed models. Murphy, Dean, and Raftery [22] extend this variable selection methodology for use with supervised learning problems, specifically with model-based discriminant analysis. These approaches formulate the problem of variable selection as a model-selection problem, whereby the features and the appropriate model are selected simultaneously.

We propose a generalized Dirichlet feature selection (GDFS) method, that is an adaptation of the above methods that is suitable for use with compositional data in a supervised learning problem. This method could also be easily adapted for use with unsupervised classification methods, such as model-based clustering. A greedy search algorithm traverses the feature space, selecting a “grouping model” at each step from a set of target generalized Dirichlet models, using the BIC for model selection and including a backwards step in the algorithm to avoid getting trapped at local maxima. At each iteration, a set of chromatographic peaks are selected as the current optimal set of “grouping variables”. Convergence is declared when no further proposed changes in the current set of selected features are accepted. The selected features are those peaks that appear to contain information about the group structure in the data, and further experimental analysis could be carried out to identify the glycan structures corresponding to these chromatographic peaks.

This method is applied to two glycan chromatography datasets; from the lung cancer study conducted by Arnold et al. [23] and from the prostate cancer study of Saldova et al. [24].

The GDFS method is compared with two well-known feature selection techniques: correlation-based feature selection (CFS) developed by Hall [26] and a recursive partitioning method (rpart) for the construction of classification trees, developed by Breiman et al. [27]. Neither method makes implicit assumptions about the distribution of the data, so both are suitable for use with compositions. Recursive partitioning builds a classification tree using a selected subset of features. It is a non-parametric method that has been used in the past for feature selection in compositional data [2,28]. Correlation-based feature selection, widely used in the machine learning community and elsewhere [29,30], is applied to a discretized form of the data. It involves a best first search over the feature space to select the set of features with the highest “merit”, a heuristic used to measure the predictive ability of a feature subset.

## Methods

Described in detail here is the proposed statistical methodology for feature selection in compositional data. This includes an introduction to the Dirichlet, beta, and generalized Dirichlet distributions, algorithmic details of the GDFS method for feature selection, a brief discussion of the two feature selection methods used for comparison and a description of the statistical classification methods employed for model validation.

Relevant information is also provided on the two glycan chromatography datasets used to test the proposed statistical methodology, along with analytical details on the glycan analysis used to collect these datasets.

### Statistical methods

### The generalized Dirichlet distribution

Connor and Mosimann [12] propose the generalized Dirichlet distribution as a more flexible extension of the ordinary Dirichlet distribution for modelling compositional data with a unit-sum constraint. This section introduces the Dirichlet distribution, followed by a description of how the Dirichlet is extended to obtain the Generalized Dirichlet model.

The Dirichlet distribution models proportional data in a simplex space. If a multivariate random vector **Y**=(*Y*_1_,*Y*_2_,…,*Y*_*p*_), such that *Y*_*j*_≥0 for *j*=1,2,…,*p* and $\overset{p}{\underset{j=1}{\sum }}Y_{j}=1$, is Dirichlet distributed with parameters ***α***=(*α*_1_,*α*_2_,…,*α*_*p*_), then the Dirichlet probability density function at **Y**=**y**_*i*_ is

(1) $$\begin{array}{ll} f(y_{i};\alpha )\;\;=\;\;\frac{1}{B(\alpha )}\overset{p}{\underset{j=1}{\prod }}y_{\mathrm{ij}}^{\alpha_{j}-1} & \; \end{array}$$

where *B*(***α***) is the multinomial beta function defined as

(2) $$\begin{array}{ll} B(\alpha )=\frac{\overset{p}{\underset{j=1}{\prod }}\Gamma (\alpha_{j})}{\Gamma (\overset{p}{\underset{j=1}{\sum }}\alpha_{j})} & \; \end{array}$$

and

(3) $$\begin{array}{ll} \Gamma (x)=\overset{\infty }{\underset{0}{\int }}t^{x-1}e^{-t}\mathrm{dt} & \; \end{array}$$

is the Gamma function.

The beta distribution is a univariate model that is a special case of the Dirichlet distribution (with *p*=2). Fitting a beta distribution to a proportional random variable *Y* is equivalent to fitting a Dirichlet distribution to (*Y*,1−*Y*), since one of the variables in the vector (*Y*,1−*Y*) is degenerate. Thus, a beta distribution has two parameters, commonly denoted (*α*,*β*), and probability density function

(4) $$\begin{array}{ll} f(y_{i};\alpha ,\beta )\;\;=\;\;\frac{1}{B(\alpha ,\beta )}\;\;y_{i}^{\alpha -1}{\left(1-y_{i}\right)}^{\beta -1}. & \; \end{array}$$

The log likelihood function for *n* observations **y**=(*y*_1_,*y*_2_,…,*y*_*n*_) of a beta distributed random variable *Y*∼beta(*α*,*β*) is given by

(5) $$\begin{array}{lll} \;\ell (\alpha ,\beta ;y)\;=\;-n\log B(\alpha ,\beta ) & +\overset{n}{\underset{i=1}{\sum }}(\alpha -1)\log y_{i}\; & \\ & +\overset{n}{\underset{i=1}{\sum }}(\beta -1)\log(1-y_{i}).\; \end{array}$$

Because of its direct relationship with the Dirichlet distribution, maximum likelihood estimates for the parameters of a beta distribution can be obtained in the same manner as for the corresponding Dirichlet distribution. The maximum likelihood estimates for these distributions do not exist in closed form, so must be obtained by numerical approximation. The fixed-point iteration method outlined by Minka [31] is used here for the numerical approximation of beta maximum likelihood estimates. Reasonable starting values can be obtained using the method of moments (Equation 15). The expectation and variance of the beta distribution are

(6) $$\begin{array}{lll} E[\;Y]\;= & \;\frac{\alpha }{\alpha +\beta }\; & \\ V[\;Y]\;= & \;\frac{E[\;Y](1-E[\;Y])}{1+\alpha +\beta }.\; \end{array}$$

Further details on parameter estimation are given in the next subsection.

Connor and Mosimann [12] derive the generalized Dirichlet distribution from their concept of neutrality for proportional vectors. A component *Y*_*j*_ of a random compositional vector **Y**=(*Y*_1_,*Y*_2_,…,*Y*_*p*_) is defined as *neutral* if it is distributed independently of the rest of the composition with *Y*_*j*_ eliminated (i.e. the remaining compositional components divided by 1−*Y*_*j*_). They extend this concept to define the idea of *c*omplete neutrality. A random compositional vector **Y**=(*Y*_1_,*Y*_2_,…,*Y*_*p*_), subject to a unit sum constraint, is said to be *completely neutral* if the elements of the vector

(7) $$\begin{array}{lll} \tilde{Y}= & \left(Y_{1},\frac{Y_{2}}{1-Y_{1}},\frac{Y_{3}}{1-Y_{1}-Y_{2}},\right)\; & \\ & \left(\ldots ,\frac{Y_{p}}{1-Y_{1}-\ldots -Y_{p-1}}\right)\; \end{array}$$

are mutually independent. The generalized Dirichlet distribution results from making the additional assumption that the marginal distributions of the elements of $\tilde{Y}$ are beta distributions. Note that the last component of $\tilde{Y}$ is degenerate since it is equal to one.

Let *S*_*j*_=*Y*_1_+*Y*_2_+…+*Y*_*j*_ be the sum of the first *j* components of **Y**, for *j*=1,2,…,*p*, and let *S*_0_=0. If $\tilde{Y}$ follows a generalized Dirichlet distribution, then **Y** is completely neutral and ${\tilde{Y}}_{j}=Y_{j}/(1-S_{j-1})∼\text{beta}(\alpha_{j},\beta_{j})$ for *j*=1,2,…,*p*−1. The probability density function for ${\tilde{Y}}_{j}$ is therefore the product of these *p*−1 marginal beta distributions, since the components of $\tilde{Y}$ are mutually independent. Making a change of variable from **Y** to $\tilde{Y}$ (see Appendix A. change of variable rule) allows the probability density function for $\tilde{Y}$ to be written in terms of the probability density function for $\tilde{Y}$, at observation *i*, as

(8) $$\begin{array}{lll} \;f(y_{i})\;\; & =\;\;f({\tilde{y}}_{i})\overset{p-1}{\underset{j=1}{\prod }}\left(\frac{1}{1-s_{i,j-1}}\right)\; & \\ \;\; & =\;\;y_{\mathrm{ip}}^{\beta_{p-1}-1}\overset{p-1}{\underset{j=1}{\prod }}\frac{1}{B(\alpha_{j},\beta_{j})}y_{\mathrm{ij}}^{\alpha_{j}-1}{\left(1-s_{i,j-1}\right)}^{\beta_{j-1}-\alpha_{j}-\beta_{j}}\; \end{array}$$

where B(*α*_*j*_,*β*_*j*_)=*Γ*(*α*_*j*_)*Γ*(*β*_*j*_)/*Γ*(*α*_*j*_+*β*_*j*_) is the beta function, *s*_*i*,*j*−1_ is the sum of the first *j*−1 compositional components for observation *i*, and $\overset{p-1}{\underset{j=1}{\prod }}1/(1-s_{i,j-1})$ is the Jacobian term resulting from the change of variable. For a full derivation of this probability density function, please refer to Appendix B. Derivation of the generalized Dirichlet probability density function. In the special case where *β*_*j*−1_=*α*_*j*_+*β*_*j*_ for *j*=1,2,…,*p*−1 and writing *α*_*p*_=*β*_*p*−1_, this model simplifies to the ordinary Dirichlet distribution given by Equation 1.

The generalized Dirichlet log likelihood for a set of *n* generalized Dirichlet samples **y**={**y**_1_,**y**_2_,…**y**_*n*_} follows from its probability density function;

(9) $$\begin{array}{lll} \ell (\theta ;y)\;\; & =\;\;\log\overset{p-1}{\underset{j=1}{\prod }}f(y_{i})\;\\ & =\overset{n}{\underset{i=1}{\sum }}(\beta_{p-1}-1)\log y_{\mathrm{ip}}-n\overset{p-1}{\underset{j=1}{\sum }}\log B(\alpha_{j},\beta_{j})\; & \\ & \;+\overset{n}{\underset{i=1}{\sum }}\overset{p-1}{\underset{j=1}{\sum }}(\alpha_{j}-1)\log y_{\mathrm{ij}}\; & \\ & \;+\overset{n}{\underset{i=1}{\sum }}\overset{p-1}{\underset{j=1}{\sum }}\;(\beta_{j-1}-\alpha_{j}-\beta_{j})\log(1-s_{i,j-1})\; \end{array}$$

where ***θ***=(*α*_1_,*β*_1_,…,*α*_*p*−1_,*β*_*p*−1_) is the generalized Dirichlet parameter vector.

Note that the ordering of generalized Dirichlet components is important. A particular ordering of compositional variables may be completely neutral, while another ordering of the same variables may not be [12]. Therefore, if a compositional vector (*Y*_1_,*Y*_2_,*Y*_3_) follows a generalized Dirichlet distribution, a permutation of its components such as (*Y*_2_,*Y*_1_,*Y*_3_) may not.

The generalized Dirichlet model is more intuitive when viewed as a tree structure. This is well explained by Null [14], who relates the generalized and nested Dirichlet distributions. Representing a generalized Dirichlet random vector as a tree structure, the compositional components are assigned to be leaves in the tree and a set of *p*−2 interior nodes are introduced. Each “nest” in the tree comprises of a leaf node (or original compositional component) and an interior node (or “nesting variable”) whose value is the sum of the leaf nodes nested below (or equivalently, one minus the sum of leaf nodes not nested beneath). The first component of the generalized Dirichlet vector is at the top of the tree structure, and successive components are nested underneath. For example, the third component is nested under the second. The nest at the bottom level of the tree consists of two leaf nodes only. The variables in each nest are beta distributed, conditional on the value of the parent (interior) node above.

**Figure 3:**
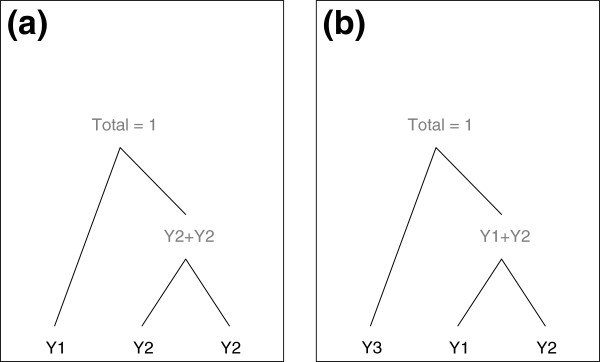
**Tree structures for the generalized Dirichlet distribution.** Figure (**a**) depicts the generalized Dirichlet tree structure of the compositional vector (*Y*_1_,*Y*_2_,*Y*_3_) following a generalized Dirichlet distribution, whereas Figure (**b**) depicts the tree structure of the compositional vector (*Y*_3_,*Y*_1_,*Y*_2_), also following a generalized Dirichlet distribution. The generalized Dirichlet models for these two nesting structures could potentially be very different. Within each nest, the variables are modelled as beta distributions, conditional on the parent node.

Figure 3 (a) illustrates this concept of a tree structure with an example, where the composition (*Y*_1_,*Y*_2_,*Y*_3_) is modelled by a generalized Dirichlet distribution. In this example, *p* = 3 so there are three leaves, *p* − 1 = 2 nests and *p* − 2 = 1 interior node in the tree. *Y*_1_ is at the top level of the tree, nested with the interior node taking the value (*Y*_2_+*Y*_3_), that is the sum of the leaf nodes nested below. The bottom nest contains the two leaf nodes, *Y*_2_ and *Y*_3_. Each nest is modelled by a beta distribution, conditional on the value of its parent node. The nest at the top level of the tree, comprising of (*Y*_1_,*Y*_2_+*Y*_3_) is modelled by a beta distribution with parameters (*α*_1_,*β*_1_) (it is not conditioned on anything, since its parent node is equal to one). The nest at the bottom level of the tree is modelled as beta, conditional on the interior node above, *Y*_2_+*Y*_3_.

The probability density function for this generalized Dirichlet model is the product of the (conditional) beta distributions for each nest in the tree,

(10) $$\begin{array}{ll} \;\left(Y_{1},Y_{2}+Y_{3}\right)∼\text{beta}(\alpha_{1},\beta_{1}) & \;\\ \left(\frac{Y_{2}}{Y_{2}+Y_{3}},\frac{Y_{3}}{Y_{2}+Y_{3}}\right)∼\text{beta}(\alpha_{2},\beta_{2}) & \; \end{array}$$

and the Jacobian term 1/(*Y*_2_+*Y*_3_) for the change of variable.

Another generalized Dirichlet model for the same components could be fitted to (*Y*_3_,*Y*_1_,*Y*_2_), as depicted in Figure 3 (b). Note that Figures 3 (a) and (b) are not the same. The probability density function for the model in Figure 3 (b) is derived from the product of

(11) $$\begin{array}{ll} \;\left(Y_{3},Y_{1}+Y_{2}\right)∼\text{beta}(\alpha_{1},\beta_{1}) & \;\\ \left(\frac{Y_{1}}{Y_{1}+Y_{2}},\frac{Y_{2}}{Y_{1}+Y_{2}}\right)∼\text{beta}(\alpha_{2},\beta_{2}) & \; \end{array}$$

and the Jacobian term 1/(*Y*_1_+*Y*_2_).

### Maximum likelihood estimation for the generalized Dirichlet distribution

The maximum likelihood estimates for a generalized Dirichlet distribution with *p* components are obtained via the estimation of parameters for the *p*−1 independent beta distributions from which the probability density function is comprised. As mentioned in the previous section, parameter estimates for the beta distribution can be obtained in the same manner as those for a Dirichlet distribution, since the beta distribution is a special case of the Dirichlet distribution.

Since maximum likelihood estimates for a Dirichlet distribution cannot be obtained in closed form, the fixed-point iteration method proposed by Minka [31] is used here to numerically approximate the beta MLEs.

For *n* observations **y**=(*y*_1_,*y*_2_,…,*y*_*n*_) of a beta distributed random variable *Y*∼beta(*α*,*β*), maximum likelihood estimates of the parameters, $\hat{\alpha }$ and $\hat{\beta }$, can be obtained by a fixed-point iteration in the following manner. At each iteration *t* of the fixed-point iteration, updated parameter estimates (*α*^*t*^,*β*^*t*^) are calculated from

(12) $$\begin{array}{ll} \Psi (\alpha^{t})\;\; & =\;\;\Psi (\alpha^{t-1}+\beta^{t-1})+\frac{1}{n}\overset{n}{\underset{i=1}{\sum }}\log\;y_{i}\; \end{array}$$

(13) $$\begin{array}{ll} \Psi (\beta^{t})\;\; & =\;\;\Psi (\alpha^{t-1}+\beta^{t-1})+\frac{1}{n}\overset{n}{\underset{i=1}{\sum }}\log\;(1-y_{i})\; \end{array}$$

where

(14) $$\begin{array}{ll} \Psi (x)=\frac{d\ln\Gamma (x)}{\mathrm{dx}} & \; \end{array}$$

is the digamma function; and then by numerical inversion of *Ψ*(*α*^*t*^) and *Ψ*(*β*^*t*^) using a Newton Raphson iteration. The fixed point iteration maximizes a lower bound on the log likelihood, and so, is sure to increase the log likelihood function at each iteration. Starting estimates for the fixed point iteration are estimated using a variant of the method of moments originally suggested by Ronning [32]. For a beta distributed random variable *Y*, with parameter vector (*α*,*β*), starting values for the parameter estimates at *t*=0 are calculated by first estimating the sum of the parameters and then obtaining estimates for each parameter,

(15) α0+β0^=E[Y](1−E[Y])V[Y]α^0=E[Y]α0+β0^β^0=E[1−Y]α0+β0^.

The relative change in parameter estimates or the relative change in the log likelihood function is often used to test for convergence during parameter estimation algorithms. However, Lindstrom and Bates [33] highlight that this provides a measure of lack of progress, rather than lack of convergence. The Aitken acceleration-based stopping criterion proposed by Böhning et al. [34] is preferred here, as a test for the convergence of a log likelihood function. The log likelihood for the beta distribution is given in Equation 5. For a linearly convergent series of log likelihood estimates, Böhning et al. suggest that an asymptotic estimate of the log likelihood at iteration ( *K*+1) is

(16) $$\begin{array}{ll} \ell_{A}^{\left(K+1\right)}=\ell^{\left(K\right)}+\frac{1}{1-c^{\left(K\right)}}\left(\ell^{\left(K+1\right)}-\ell^{\left(K\right)}\right). & \; \end{array}$$

where *ℓ*^(*K*)^ and *c*^(*K*)^ denote the log likelihood and the Aitken acceleration at any iteration *K*, respectively, where the Aitken acceleration is defined by

(17) $$\begin{array}{ll} c^{\left(K\right)}=\frac{\ell^{\left(K+1\right)}-\ell^{\left(K\right)}}{\ell^{\left(K\right)}-\ell^{\left(K-1\right)}}. & \; \end{array}$$

Lindsay [35] suggests that the optimization algorithm should be terminated when the difference in the projected and current log likelihoods is less than than some specified tolerance level,

(18) $$\begin{array}{ll} \ell_{A}^{\left(K+1\right)}-\ell^{\left(K+1\right)}<\text{tol}. & \; \end{array}$$

A similar criterion, proposed by McNicholas et al. [36] is used here as a stopping criterion:

(19) $$\begin{array}{ll} \ell_{A}^{\left(K+1\right)}-\ell^{\left(K\right)}<\text{tol}. & \; \end{array}$$

A tolerance level of 0.003 was used here, as it appeared sufficient for convergence upon examination of log likelihood sequences.

The parameter vector for a generalized Dirichlet distribution for a composition with *p* components is written (*α*_1_,*β*_1_,*α*_2_,*β*_2_,…,*α*_*p*−1_,*β*_*p*−1_), where each pair (*α*_*j*_,*β*_*j*_) are the parameters for the beta distribution of the *j*th nest in the tree structure, with *j*=1 corresponding to the top level and *j*=*p*−1 corresponding to the bottom level.

#### Feature selection using the generalized Dirichlet distribution

This section describes the GDFS method for compositional data. Let **Y**=(*Y*_1_,*Y*_2_,…,*Y*_*p*_) denote a unit-sum compositional random vector. Let *Z* be a random variable indicating the group to which an observation **Y**=**y**_*i*_ belongs, so that *z*_*i*_=*g* if **y**_*i*_ belongs to group *g*. Components in **Y** that contain group information will therefore be dependent on *Z*.

The compositional variables **Y** are partitioned into three sets, as follows: 

1. **Y**^(*c*)^: variables that contain group information (that are currently in the grouping model)

2. **Y**^(*o*)^: variables that do not contain group information (that are currently omitted from the grouping model)

3. *Y*^(*p*)^: the proposal variable (the variable proposed for addition or removal from the current grouping set, **Y**^(*c*)^)

The objective of feature selection is to choose the set of features, compositional components in this case, that differ across known groups in the data. Therefore, the final objective is to find the optimal partition {**Y**^(*c*)^,**Y**^(*o*)^} of grouping and non-grouping variables. A greedy algorithm is used to search efficiently through the space of all possible partitions of $\tilde{Y}$ for the optimal partition. At each iteration of the greedy algorithm, the current state is defined by some partition of $\tilde{Y}$, and it is proposed to add or remove a variable to or from the grouping set **Y**^(*c*)^. The decision to accept or reject this proposal is made by examining whether the proposal variable, *Y*^(*p*)^ contains group information or not; that is, whether or not it depends on the group indicator variable *Z*. The probability density function at an observed value of **Y**=**y**_*i*_ can be factorized into parts corresponding to the partition of **x** using standard laws of conditional probability,

(20) $$\begin{array}{lll} f(y_{i}\left(\right|z_{i})\;\; & =\;\;f(y_{i}^{\left(c\right)},y_{i}^{\left(p\right)},y_{i}^{\left(o\right)}|z_{i})\; & \;\\ \;\; & =\;\;f(y_{i}^{\left(c\right)},y_{i}^{\left(p\right)}|z_{i})f(y_{i}^{\left(o\right)}\left(\right|y_{i}^{\left(p\right)},y_{i}^{\left(c\right)})\; & \;\\ \;\; & =\;\;\underset{\left(\mathrm{iii}\right)}{\underset{︸}{f(y_{i}^{\left(c\right)}|z_{i})}}\underset{\left(\mathrm{ii}\right)}{\underset{︸}{f(y_{i}^{\left(p\right)}|y_{i}^{\left(c\right)},z_{i})}}\underset{\left(i\right)}{\underset{︸}{f(y_{i}^{\left(o\right)}\left(\right|y_{i}^{\left(p\right)},y_{i}^{\left(c\right)})}}.\; \end{array}$$

When proposing to add (remove) *Y*^(*p*)^ to (from) the grouping model, two models can be considered; the first is a model where part (*ii*) of Equation (20) depends on *Z*, indicating that *Y*^(*p*)^ contains group information. The second is a model where the density function in part (*ii*) does not depend on *Z*, indicating that the proposal variable, *Y*^(*p*)^, does not contain group information and should be excluded from the grouping set.

The proposed GDFS method chooses a set of “grouping variables” via the construction of a generalized Dirichlet model. If it is assumed that the partition {**Y**^(*c*)^,*Y*^(*p*)^,**Y**^(*o*)^} follows a generalized Dirichlet distribution (and hence the ordering of variables is important), then the distribution of ${\tilde{Y}}^{\left(p\right)}=Y^{\left(p\right)}/(1-S^{\left(c\right)})$ is a beta distribution. Denoting the probability density function of ${\tilde{Y}}^{\left(p\right)}$ by $f_{{\tilde{Y}}^{\left(p\right)}}$, the conditional distribution of *Y*^(*p*)^ given **Y**^(*c*)^ and *Z* is derived using the change of variable rule in Appendix A. change of variable rule, as

(21) $$\begin{array}{ll} f(y_{i}^{\left(p\right)}|{\text{y}}_{i}^{\left(c\right)},z_{i})\;\;\; & =\;\;\;f_{{\tilde{Y}}^{\left(p\right)}}({\tilde{y}}_{i}^{\left(p\right)}|z_{i})\left(\frac{1}{1-s_{i}^{\left(c\right)}}\right),\; \end{array}$$

which is independent of **Y**^(*c*)^ since (**Y**^(*c*)^,*Y*^(*p*)^,**Y**^(*o*)^) is completely neutral. Therefore, the density of the proposal variable *Y*^(*p*)^ is the product of a beta distribution and the Jacobian term, 1/(1−*S*^(*c*)^).

Interestingly enough, the notion of partitioning variables into independent subspaces of components has previously been considered in independent subspace analysis (ISA), which has been applied to feature extraction problems in the past [37].

#### Proposal to add a component to **Y**^(*c*)^

At every iteration of the greedy search algorithm, it is proposed to add a component to the grouping set, considering each of the currently omitted components. The decision of whether a proposed component *Y*^(*p*)^ contains group information is made by comparing a grouping and non-grouping model for *Y*^(*p*)^. In the grouping model, *Y*^(*p*)^ is dependent on *Z*, and in the non-grouping model it is not.

In terms of Equation (20), these models will be identical except for part (*ii*). Thus, the proposal to add *Y*^(*p*)^ to the set of grouping variables is considered via a comparison of a grouping and non-grouping model, denoted *M*_*GR*_ and *M*_*NGR*_ respectively, where

(22) $$\begin{array}{cc} M_{\mathrm{GR}} & :\;f_{{\tilde{Y}}^{\left(p\right)}}\left({\tilde{y}}_{i}^{\left(p\right)}\left|z_{i}\right)\right)\left(\frac{1}{1-s_{i}^{\left(c\right)}}\right)\\ M_{\mathrm{NGR}} & :\;\;\;f_{{\tilde{Y}}^{\left(p\right)}}\left({\tilde{y}}_{i}^{\left(p\right)}\right)\left(\frac{1}{1-s_{i}^{\left(c\right)}}\right).\; \end{array}$$

The Jacobian term, 1/(1−*si*(*c*)), can be neglected in this comparison since it is common to both models. Since ${\tilde{Y}}^{\left(p\right)}$ is beta distributed, the fitted grouping and non-grouping models for the proposal variable will be:

(23) $$\begin{array}{lll} M_{\mathrm{GR}} & :\;Y^{\left(p\right)}/(1-S^{\left(c\right)})\left|\right)\left(Z=g\right)∼\text{beta}(\alpha_{g},\beta_{g})\; & \;\\ M_{\mathrm{NGR}} & :\;\;Y^{\left(p\right)}/(1-S^{\left(c\right)})∼\text{beta}(\alpha ,\beta )\; \end{array}$$

The parameters for the grouping model are group dependent and must be estimated separately for each group.

If the grouping model for the proposal variable provides a better fit than the non-grouping model, then the proposal variable should considered for addition to the grouping set, **Y**^(*c*)^. Note that if it is added to **Y**^(*c*)^, it should be added to the end of **Y**^(*c*)^ rather than the beginning, to indicate that it is nested underneath variables that were added before it. This is necessary for the model to be a generalized Dirichlet distribution, considering the specified grouping and non-grouping model structure.

#### Proposal to remove a component from **Y**^(*c*)^

A proposal to remove a variable from the grouping model is also included at each iteration. This could potentially reduce the possibility of getting stuck at a local maxima. The decision of whether to remove a proposed component *Y*^(*p*)^ from the grouping set **Y**^(*c*)^ is made by comparing a grouping and non-grouping model for *Y*^(*p*)^. In the grouping model, *Y*^(*p*)^ depends on the group information vector *Z*, and in the non-grouping model it does not.

For the remove step, the “grouping model” considered is the generalized Dirichlet model fitted to the current set of grouping variables, denoted here as **Y**^(*c*,*p*)^. This notation is used here to indicate that *Y*^(*p*)^ is included amongst the grouping set, in the ordering specified by the currently fitted generalized Dirichlet model for the grouping set. It differentiates from the notation (**Y**^(*c*)^,*Y*^(*p*)^), which indicates that component *Y*^(*p*)^ is definitely at the end of the vector (i.e. in the bottom nest of the generalized Dirichlet tree structure). If **Y**^(*c*,*p*)^ follows a generalized Dirichlet distribution, this does not imply that (**Y**^(*c*)^,*Y*^(*p*)^) is also generalized Dirichlet distributed. When proposing to remove a component from the grouping set, the generalized Dirichlet model fitted to **Y**^(*c*)^ must also be considered in the comparison of grouping and non-grouping models. This is because removing the proposal variable from the generalized tree structure could result in a different generalized Dirichlet tree structure for the set of grouping variables.

In terms of the second line of Equation 20, the density function for the grouping model can be factorized as

(24) $$\begin{array}{ll} f(y_{i}\left(\right|z_{i})\;\; & =\;\underset{\left(a\right)}{\underset{︸}{f({\text{y}}_{i}^{\left(c,p\right)}|z_{i})}}\underset{\left(b\right)}{\underset{︸}{f(y_{i}^{\left(o\right)}\left(\right|y_{i}^{\left(p\right)},y_{i}^{\left(c\right)})}}.\; \end{array}$$

For the remove step, the density for the non-grouping model can be factorized as in the third line of Equation 20, where the proposal variable is not dependent on *Z*,

(25) $$\begin{array}{ll} f(y_{i}\left(\right|z_{i})\;\; & =\;\underset{\left(a\right)}{\underset{︸}{f({\text{y}}_{i}^{\left(c\right)}|z_{i})f(y_{i}^{\left(p\right)}|{\text{y}}_{i}^{\left(c\right)})}}\underset{\left(b\right)}{\underset{︸}{f(y_{i}^{\left(o\right)}\left(\right|y_{i}^{\left(p\right)},y_{i}^{\left(c\right)})}}.\; \end{array}$$

Component (b) of the grouping and non-grouping models are the same and can be omitted from a comparison of the two models. Neglecting component (b) results in a simplified comparison of a grouping and non-grouping model for *Y*^(*p*)^, denoted *M*_*GR*_ and *M*_*NGR*_ respectively, where

(26) $$\begin{array}{lll} M_{\mathrm{GR}}: & \;f\left({\text{y}}_{i}^{\left(c,p\right)}\left|\right)z_{i}\right)\; & \;\\ M_{\mathrm{NGR}}: & \;f\left({\text{y}}_{i}^{\left(c\right)}\left|\right)z_{i}\right)f_{{\tilde{Y}}_{p}}\left({\tilde{y}}_{i}^{\left(p\right)}\right)\left(1/(1-s_{i}^{\left(c\right)})\right)\; \end{array}$$

where $1/(1-s_{i}^{\left(c\right)})$ is the Jacobian term resulting from the change of variable from $y_{i}^{\left(p\right)}$ to ${\tilde{y}}_{i}^{\left(p\right)}$ (see Equation 21). Letting **A**^(*j*)^ denote the parameter vector for the generalized Dirichlet model fitted to the compositional components in set *j*, the grouping and non-grouping models to be fitted at the remove step may be written,

$$\begin{array}{lll} M_{\mathrm{GR}}: & \;{\text{y}}_{i}^{\left(c,p\right)}\left|\right)z_{i}∼\mathrm{GD}({\text{A}}_{g}^{\left(c,p\right)})\; & \;\\ & \; \end{array}$$

(27) $$\begin{array}{lll} M_{\mathrm{NGR}}: & \;{\text{y}}_{i}^{\left(c\right)}\left|\right)z_{i}∼\mathrm{GD}({\text{A}}_{g}^{\left(c\right)})\; & \;\\ & \;\;y_{i}^{\left(p\right)}|{\text{y}}_{i}^{\left(c\right)}∼\text{beta}(\alpha ,\beta ),\; \end{array}$$

and the probability density functions for each are calculated from Equation 26. For the set of grouping variables, the parameter vector is indexed by *g* to indicate that they are estimated separately for each group *g*. At every remove step, each of the components currently in the grouping model are considered as remove proposals. If the grouping model provides a better fit than the non-grouping model, this can be considered as evidence for retaining the proposal variable in the grouping set. If the converse is true, there is evidence for removing the proposal variable from the grouping set.

#### Selected feature model

When the partition {**Y**^(*c*)^,**Y**^(*o*)^} is found that is considered to be optimal, the “grouping model” is the generalized Dirichlet model currently fitted to **Y**^(*c*)^. Note that this is equivalent to fitting a generalized Dirichlet model to (**Y**^(*c*)^,1−*S*^(*c*)^), since the component 1−*S*^(*c*)^ is degenerate (it is equal to the sum of the omitted variables).

The parameters for this grouping model should be estimated separately for each group, since these are the components that are considered to be dependent on *Z*.

#### Bayesian Information Criterion (BIC)

The Bayesian Information Criterion (BIC) is a model selection criterion that was proposed by Schwarz [21] and was used by Raftery and Dean [19] for model selection in variable selection for model-based clustering. The BIC is also used here for model comparison. For a beta distribution, the BIC is given by

(28) $$\begin{array}{ll} 2\ell (\hat{\alpha },\hat{\beta };y)-2\log n & \; \end{array}$$

where $\ell (\hat{\alpha },\hat{\beta };y)$ is the log likelihood function given in Equation 5, evaluated at the maximum likelihood estimates of the parameters, $\alpha =\hat{\alpha }$ and $\beta =\hat{\beta }$. The BIC prevents model overfitting by using a penalty for model complexity, number of parameters× log*n*. In comparing two models, that with the larger BIC is preferable.

The BIC in Equation 28 is used to compare beta distributions for the proposal variable in the grouping and non-grouping models specified in Equation 23. The BIC for the grouping model is computed as the sum of the BIC values obtained from fitting a beta distribution to each group. Then the decision of whether the proposal variable *Y*^(*p*)^ contains group information is made by examination of the difference in BIC for the grouping and non-grouping models.

(29) $$\begin{array}{ll} {\text{BIC}}_{\text{diff}}={\text{BIC}}_{\text{GR}}-{\text{BIC}}_{\text{NGR}} & \; \end{array}$$

A positive value for BIC_diff_ provides evidence in favour of grouping model, *M*_*GR*_, over the non-grouping model, *M*_*NGR*_. The larger the difference in BIC, the more statistical evidence there is in favour of including *Y*^(*p*)^ in the set of grouping variables.

For a generalized Dirichlet distribution, the BIC is calculated by

(30) $$\begin{array}{ll} 2\ell (\hat{\theta };y)-2(p-1)\log n & \; \end{array}$$

where $\ell (\hat{\theta };y)$ is the log likelihood function of the generalized Dirichlet distribution given by Equation (9), evaluated at $\theta =\hat{\theta }$, the maximum likelihood estimate for the parameter vector, 2(*p*−1) is the number of estimated parameters, and *n* is the number of samples. This BIC can be used to compare generalized Dirichlet distributions fitted to different orderings/permutations of the grouping variable set **Y**^(*c*)^, as outlined in the following section.

#### Algorithm outline

This section outlines the proposed feature selection algorithm for compositional data. The model is initialized by adding two compositional components to the grouping model. The algorithm iterates over three steps until convergence, the first step being the proposal to add a component to the grouping model. Greedy searches can get trapped at local maxima, so the second and third steps are included to avoid this. The second step is a proposal to remove a component from the grouping model, while the third is a proposal to permute the order of nesting in the generalized Dirichlet grouping model i.e. to permute the ordering of components in **Y**^(*c*)^. Each step proposes a “move” that is either accepted or rejected. The algorithm terminates when an add, remove, and permute proposal are rejected in succession. 

1. INITIALIZATION: Initially assign all variables to the non-grouping set, and then add a single variable to the grouping set. The decision of which variable to add is made via a comparison of BIC differences for grouping and non-grouping models for each variable. The variable with the maximum BIC difference is added to the grouping model. If all of the BIC differences are negative, the variable with the least negative BIC difference is added. Add a second variable to the grouping model in a similar manner. If this second add move is not made, the algorithm will terminate after the first iteration if the BIC difference was negative for the first variable added (as the variable will be removed and no further add moves will be made).

2. ADD STEP: Propose to add a variable to the grouping model. The decision of whether to add a variable to the grouping model is made via a BIC comparison for grouping and non-grouping models for each variable in **Y**^(*o*)^, the non-grouping set. If any of the BIC differences for these models is positive, add the variable with the largest positive BIC difference to the grouping set. A positive BIC difference provides evidence that a variable contributes group information to the model. If all BIC differences are negative, reject the proposal to add a variable to the grouping model. If the proposal to add a variable is accepted, this variable is added to the end of **Y**^(*c*)^. This means that it will be located in the bottom nest of the generalized Dirichlet tree structure fitted to the grouping variables.

3. REMOVE STEP: Propose to remove a variable from the grouping model. The decision of whether to remove a variable is made via a BIC comparison for grouping and non-grouping models for each variable currently included in the grouping set, **Y**^(*c*)^. A negative BIC difference provides evidence that a variable does not contribute group information to the model. If the BIC difference is negative for any of these variables, remove the variable with the largest negative BIC difference from the grouping set, and add it to the non-grouping set **Y**^(*o*)^. If all BIC differences are positive, reject the proposal to remove a variable from the grouping model.

4. PERMUTE STEP: If there are two or more variables in the grouping model, propose to permute order of the components in **Y**^(*c*)^. Permuting the order of **Y**^(*c*)^ will change the generalized Dirichlet tree structure and will result in a different generalized Dirichlet model for the set of grouping variables. Set MAXPERM to be the maximum number of permutations to be considered at any iteration. Used here was a maximum of 60 permutations. Setting a maximum is necessary for computational efficiency, because if there are *m* variables in the grouping set, the number of possible permutations is *m*! and increases quickly as more variables are added to the grouping set. The number of permutations, NPERM, considered at a particular iteration is defined as the minimum of *m*! and MAXPERM. Calculate the BIC for the currently fitted generalized Dirichlet model, and then fit generalized Dirichlet models to NPERM randomly generated permutations of the grouping variables, **Y**^(*c*)^. Let the permutation with the largest BIC be the proposal model. If the proposal model has a larger BIC than the current generalized Dirichlet model, let the proposal model be the current model for the grouping variables. As an example, if (*Y*_2_,*Y*_4_) is the current grouping set, evaluate the BIC of the current generalized Dirichlet model, fitted to (*Y*_2_,*Y*_4_,1−*Y*_2_−*Y*_4_). Consider the permutation (*Y*_4_,*Y*_2_). Evaluate the BIC of a generalized Dirichlet distribution fitted to (*Y*_4_,*Y*_2_,1−*Y*_2_−*Y*_4_). If this is larger than the current BIC, then let the current grouping set be (*Y*_4_,*Y*_2_).

5. TERMINATION: Iterate over steps 2 to 4 until an add, remove, and permute proposal are rejected in succession. The selected components **Y**^(*c*)^ at this point, and their selected ordering, are the optimal feature set to be returned from the algorithm.

**Figure 4:**
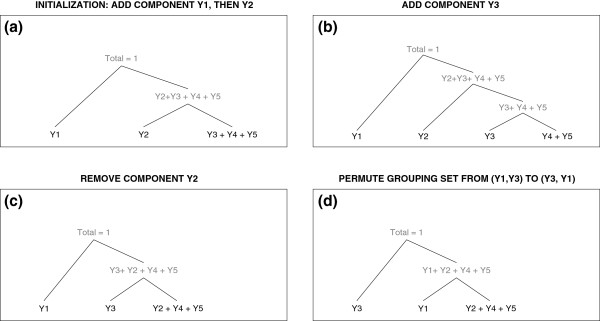
**Example of tree structures obtained over one iteration of the proposed feature selection algorithm.** Possible generalized Dirichlet tree structures that could be obtained from the initialization step and one iteration of the proposed feature selection algorithm, for a composition **Y** with five components. Figure (**a**) shows a possible tree structure that could be obtained from the initalization step, where component *Y*_1_ is added first, followed by *Y*_2_. In the subsequent figures, one possible outcome of an iteration over the GDFS algorithm is depicted. Component *Y*_3_ is added in Figure (**b**), component *Y*_2_ is removed in Figure (**c**). Figure (**d**) shows the final step for this iteration, where the current grouping model **Y**^(*c*)^=(*Y*_1_,*Y*_3_) is permuted to **Y**^(*c*)^=(*Y*_3_,*Y*_1_). This would occur if the BIC of the fitted generalized Dirichlet model for (*Y*_3_,*Y*_1_) gave a higher BIC than that for the model fitted to (*Y*_1_,*Y*_3_).

Figure 4 demonstrates a possible initialization step as well as an iteration over an add, remove, and permute step of the above algorithm, for a composition **Y** with five components. Depicted are some possible generalized Dirichlet tree structures that could be obtained if the proposals to add, remove, and permute are all accepted.

### Competing feature selection methods

Two alternative methods for feature selection were applied to the glycan chromatography data for comparison with the proposed GDFS method. The first is the correlation-based feature selection (CFS) algorithm developed by Hall [26], while the second is a classification tree method developed by Breiman et al. [27]. These methods do not make implicit assumptions about the distribution of the data, and so they are both suitable for compositional data analysis. A brief outline of each method is provided here.

**CFS**: Hall [26] proposed a correlation-based feature selection method, involving a best-first search over the feature space, evaluating subsets of features based on their “merit”. Continuous features are first discretized using the MDL method of Fayyad and Irani [38]. The degree of association between each pair of features, as well as the association between each feature with the class variable, is calculated by symmetrical uncertainty. That is, for any two nominal variables *X* and *Y*,

(31) $$\begin{array}{ll} \text{symmetrical uncertainty}=2\times \left(\frac{H(Y)-H(Y|X)}{H(Y)+H(X)}\right) & \; \end{array}$$

where

(32) $$\begin{array}{ll} H(Y)\;\; & =\;\;-\underset{y\in Y}{\sum }p(y)\underset{2}{\log}p(y)\; \end{array}$$

is the entropy of *Y*, with the proportion of observations at level *y* of *Y* denoted by *p*(*y*) and

(33) $$\begin{array}{ll} H(Y|X)\;\; & =\;\;-\underset{x\in X}{\sum }p(x)\underset{y\in Y}{\sum }p(y|x)\underset{2}{\log}p(y|x)\; \end{array}$$

is the conditional entropy of *Y* given *X*. Cover and Thomas [39] provide a comprehensive review of information theory, including a detailed discussion on entropy. Here, *p*(*y*|*x*) is the proportion of observations observed at level *y* of *Y*, within level *x* of *X*.

The “merit” is a heuristic measuring the predictive ability of a feature set, divided by the redundancy of that feature set. For a selected set of features *S*, the merit is calculated by

(34) $$\begin{array}{ll} {\text{Merit}}_{s}\;\;=\;\;\frac{k\bar{r_{\mathrm{cf}}}}{\sqrt{k+k(k-1)\bar{r_{\mathrm{ff}}}}} & \; \end{array}$$

where $\bar{r_{\mathrm{ff}}}$ is the average symmetrical uncertainty between all pairs of features in *S*, $\bar{r_{\mathrm{cf}}}$ is the average symmetrical uncertainty between the features and the class variable, and *k* is the number of features in *S*. The idea behind using the merit as a heuristic for feature selection, is that a “good” set of features will be highly correlated with the class variable, but not highly correlated with each other.

The best-first search algorithm starts at an empty node (corresponding to an empty feature set). Predecessors of the current node, S, are the set of nodes generated by adding each of the currently omitted features to the feature set at the current node. The algorithm terminates when five consecutive fully expanded nodes have provided no improvement in the merit score, or when all nodes have been visited (typically only happens where the feature space is of low dimensionality). In the case where the merit score does not improve from zero, an empty feature set should be returned. Pseudocode for the correlation based feature selection algorithm is provided in Appendix C. Construction of classification trees using recursive partitioning. Further technical details are given by Hall [26].

To obtain measures of classification performance for the chosen feature set, Dirichlet distributions are fitted to each group, using the selected feature set. Posterior probabilities are calculated using a maximum a posteriori (MAP) classification rule. Further details are provided in the section below.

**rpart**: Baxter and Jackson [2], Ranganathan and Borges [1]*], and Vermeesch [*[28] use classification tree methods for compositional data analysis. The method used here is the same as that applied by Baxter and Jackson [2], a recursive partitioning algorithm developed by Breiman et al. [27]. Model fitting is carried out by the **rpart** package in R [40]. A brief summary of the methodology employed by **rpart** is included in Appendix B. Derivation of the generalized Dirichlet probability density function. More technical details of the recursive partitioning algorithm and the software implementation may be found in the technical report by Therneau and Atkinson [41].

#### Classification and selection bias

Each of the feature selection methods described above choose a set of “grouping” features and return a set of posterior group probabilities for the observations in **Y**. Statistical classification is used to measure how well a selected feature set separates the set of known groups, to determine whether the feature selection algorithm has chosen a “good” feature set. Maximum a posteriori (MAP) classifications, calculated from the selected feature set, are used assign observations to groups.

In feature selection, where a classification rule is formed on the selected feature set, using the same samples that were used to select those features, there are two major sources of bias that can be introduced. The first is classification bias, where a classification rule is trained and tested on the same dataset. The second is selection bias, arising where the classification rule is tested on observations that were used to select the set of features that form the classification rule. Ambroise and McLachlan [42] review this problem and suggest two alternative means of overcoming such bias in feature selection problems. Their recommendation of calculating the misclassification error external to the feature selection process is followed here. Leave-one-out (LOO) cross-validation is used during the feature selection process to avoid the introduction of selection bias. Statistical classifications obtained during the GDFS method are carried out in the following manner: For each observation *j*, 

1. Observation *j* (test data) is omitted from the data, and feature selection is carried out on the remaining observations (training data).

2. For each group of observations in the training data, a generalized Dirichlet distribution is fitted to the selected feature set, **Y**^(*c*)^.

3. Using the fitted generalized Dirichlet models fitted to **Y**^(*c*)^ for each group defined by *Z*, posterior group probabilities are calculated for observation *i* using Bayes rule:

(35) $$\begin{array}{ll} \;P(g\left|\right)y_{i}^{\left(c\right)})\; & =\;\frac{\tau_{g}\;f(y_{i}^{\left(c\right)}\left|\right)z_{i}=g)}{\overset{G}{\underset{j=1}{\sum }}\tau_{j}\;f(y_{i}^{\left(c\right)}\left|\right)z_{i}=j)}\text{for}g=1,2,\ldots ,G\; \end{array}$$

where *G* is the number of groups in the data.

4. Observation *i* is classified to some group *g* using the MAP classification rule, so observation **y**_*i*_ is assigned to group *g* if

(36) $$\begin{array}{ll} g={\text{argmax}}_{j}P(j\left|\right)y_{i}^{\left(c\right)})\text{for}\;j=1,2,\ldots ,\mathrm{G.} & \; \end{array}$$

Because the glycan chromatography datasets are from observational studies, the number of patients in each group is not representative of the general population, and so we assume here that the prior probability of group membership is equal across all groups. Selection bias is avoided, since observation *j* is not used in the selection of the feature set that it is classified under.

The above procedure is repeated for each observation *i*. The cross-validated error rate is then calculated as the proportion of observations that were incorrectly classified by the above method.

For correlation-based feature selection, classifications are obtained in the same manner, except that the assumption is made that the selected grouping features are distributed according to a Dirichlet distribution, rather than a generalized Dirichlet distribution. Then in step 2, a Dirichlet distribution is fitted to the grouping features with observation *i* omitted, and at steps 3, the probability density functions in Equation 35 correspond to the Dirichlet rather than generalized Dirichlet distributions fitted to each group.

For the recursive partitioning (rpart) method the same steps for the classification of observations are used, with the exception of steps 2 and 3. Posterior probabilities for a classification tree are defined within the tree construction process. Each leaf in the tree has an associated set of posterior probabilities for each group, corresponding to the proportions of observations in the training data that belonged to each group, that were classified to that leaf node. Posterior group probabilities are obtained for a new observation by dropping it down the tree until it reaches a leaf node. The posterior group probabilities for that observation are the class proportions assigned to that leaf during the building of the tree. These probabilities are used in place of those obtained from steps 2 and 3 in the above algorithm.

#### Measures of classification performance

Classification results for each feature selection method are reported via a cross-tabulation of the true and predicted group memberships. Also included are the following measures of classification performance:

##### 

**Cross-validation error:** the proportion of observations incorrectly classified, calculated by the proportion of observations on the off-diagonal of the confusion matrix.

##### 

**kappa:** Cohen’s kappa statistic [43] is another measure of class agreement, recording the proportion of observations correctly classified, corrected for classification by chance. It is calculated as *κ*=(*O*−*E*_*chance*_)/(1−*E*_*chance*_), where *O* is the observed or actual proportion of observations correctly classified and *E*_*chance*_ is the expected proportion of observations that would be classified correctly by chance. If all observations are correctly classified, then *κ*=1. If the classification performance is no better than what one could expect by chance, *κ*≤0.

##### 

**Sensitivity:** the proportion of true positives. In assessing the diagnostic accuracy of a test, the sensitivity is measured by the proportion of disease cases correctly diagnosed by the test. For life threatening diseases, a test with high sensitivity is vitally important.

##### 

**ROC curves:** ROC curves allow for the visualization of the true positive rate (sensitivity) against the false positive rate (1 - specificity) of a classifier, where the probability threshold for classification is varied over the interval [0,1]. ROC curves and their corresponding AUC (area under the ROC curve) values are commonly reported in the biological sciences, so these are also included as performance measures for each of the feature selection methods.

##### 

**AUC:** area under a ROC curve. Values range between 0 and 1, with larger values indicating better classification performance. Fawcett [44] gives a useful interpretation of the AUC, as being equivalent to the probability that a randomly chosen disease case will be ranked higher than a randomly chosen control, by the classifier.

#### Software

All statistical analyses were carried out using R version 2.13 [40]. ROC curves were constructed and AUC values estimated using the ROCR package in R [45], while classification trees were fitted using the rpart package.

### *N*-glycan analysis

*N*-glycan analysis was carried out on two datasets, from a lung cancer study and a prostate cancer study. Samples were obtained with ethical consent from their respective sources. The glycan analysis was carried out using HILIC. Details on experimental conditions are provided below.

#### Lung cancer serum samples

Serum samples from preoperative patients diagnosed with lung cancer and cancer-free healthy volunteers were obtained from Fox Chase, Cancer Center, Philadelphia, USA under IRB approved protocols. They were from both males and females. Patient sera (20 from each stage - I, II, IIIA, IIIB, IV) were examined alongside 84 age-matched control sera from donors who did not have cancer.

*N*-glycan analysis was carried out by HILIC flouresence using a 60-minute method. The glycan HILIC profiles produced were integrated over a set of 17 glycan peaks, resulting in a 17 part compositional vector for each observation. An example of one such glycan HILIC profile is shown in Figure 1. Further details on the analysis may be found in Arnold et al. [23].

#### Prostate cancer serum samples

Samples were collected with consent from prostate cancer patients before undergoing radical prostatectomy and from men with benign prostate hyperplasia (BPH) following a standard operating procedure, which is part of the Prostate Cancer Research Consortium BioResource. Ethical consent was granted from respective Hospital ethics committee of the consortium. Blood samples (10 mL) were collected into anticoagulant-free tubes. Samples were coded and transported on ice to the laboratory. The tubes were centrifuged at 2500 rpm at 20°C for 10 min. within a 30 min. time frame. Serum from each patient sample was then collected, aliquoted, and stored at –80°C until time of analysis. Each serum sample underwent no more than three freeze/thaw cycles prior to analysis. *N*-glycan analysis was carried out by HILIC fluorescence using a 60 minute method. The glycan HILIC profiles produced were integrated over a set of 24 glycan peaks, resulting in a 24 part compositional vector for each observation in our data. An example of one such glycan HILIC profile is shown in Figure 2. Further details on the data collection and analysis may be found in Saldova et al. [24].

#### *N*-glycan analysis for patients with lung and prostate cancer

*N*-glycans were released from serum using the high-throughput method described by Royle et al. [9]. Briefly, serum samples were reduced and alkylated in 96-well plates, and then they were immobilized in SDS-gel blocks and were washed. The *N*-linked glycans were released using peptide *N*-glycanase F (1000 U/mL; EC 3.5.1.52) as described previously [46,47]. Glycans were fluorescently labeled with 2-aminobenzamide (2 AB) by reductive amination [46] (LudgerTag 2-AB labeling kit LudgerLtd., Abingdon, UK). HILIC was performed using a TSK-Gel Amide-80 column (Anachem, Luton, Bedfordshire, UK) on a 2695 Alliance separation module (Waters, Milford, MA) equipped with a Waters temperature control module and a Waters 2475 (lung cancer data) or 474 (prostate cancer data) fluorescence detector. Solvent A was 50 mM formic acid which was adjusted to pH 4.4 with ammonia solution. Solvent B was acetonitrile. The column temperature was set to 30°C. Gradient conditions were as follows: 60 min. method - a linear gradient of 35 to 47% solvent A over 48 min. at a flow rate of 0.8 mL/min, followed by 1 min. at 47 to 100% A and 4 min. at 100% A, returning to 35% A over 1 min., and then finishing with 35% A for 6 min. [9]. Samples were injected in 80% (lung cancer data) or 65% (prostate cancer data) acetonitrile. Fluorescence was measured at 420 nm with excitation at 330 nm. Royle et al. [9] described the *N*-glycosylation in human serum in detail and showed that there are 117 *N*-glycans present.

This method enables the analysis of glycan isoforms based on sequence and linkage (for example, core *α*1-6 fucosylation can be distinguished from *α*1-3 linked outer arm fucosylation). Glycan size and linkage result in a specific elution position that can be converted to glucose units (GUs) using a dextran hydrolysate standard ladder [9].

Glycan HILIC peaks were integrated and relative peak areas calculated using the Waters Empower 3 chromatography data software. Thus, each serum sample generates a data observation, consisting of the set of relative proportional peaks areas from a glycan HILIC profile.

## Results and discussion

The proposed GDFS method was applied to the lung and prostate cancer datasets. The results are compared with those of two well-established feature selection methods; correlation based feature selection and recursive partitioning (rpart).

For the lung cancer dataset, the group structure is redefined as control versus cancer. All three feature selection methods perform reasonably well. The GDFS method gives the best performance with a classification rate of approximately 75% and an AUCvalue of 0.83.

Two different group structures are considered for the prostate cancer dataset. Feature selection was carried out on the data, with cases grouped as control or prostate cancer, to determine whether any features could have diagnostic value for prostate cancer. However, none of the feature selection methods were successful at classification. CFS chooses no features most of the time, while the other two methods produce feature selection with very poor classification performance.

The other research question of interest for the prostate cancer dataset was whether glycosylation could be used as a marker of disease progression. Thus, feature selection was also applied to the prostate cancer samples, classified as into Gleason 5 and Gleason 7 cases. A Gleason score of 7 indicates a more advanced cancer of the prostate.

The results from these analyses are shown here. Following the discussion of these feature selection results is a note on the computational complexity of the GDFS method used and its behaviour in moving to higher dimensions.

### Lung cancer data

Jemal et al. [48] reported that lung cancer is the most common cancer globally, responsible for 1.4 million deaths each year. It has a very poor 5-year survival rate of 8–16%, that is mainly attributable to the disease only presenting symptoms when it reaches an advanced stage [49]. Early stage detection of lung cancer could greatly improve the outlook of patients. Ghosal et al. [49] highlight that, in an attempt to reduce the mortality rates of this disease, much research has been carried out in the area of lung cancer screening and biomarker discovery. Serum biomarkers would provide a non-invasive method for cancer diagnosis. However, although a number of potential biomarkers have been identified, none to-date seem to have adequate sensitivity, specificity or reproducibility to be used in clinical diagnostics.

Arnold et al. [23] conducted a study to investigate alterations or irregularities that occur in the serum *N*-glycome of lung cancer patients. The main objective was to identify a set of glycan structures that have biomarker potential.

Feature selection was carried out on the glycan chromatography dataset from this study using the proposed GDFS method, as well as two competing methods, CFS and rpart. The results are compared here.

#### Feature selection for lung cancer data

**Figure 5:**
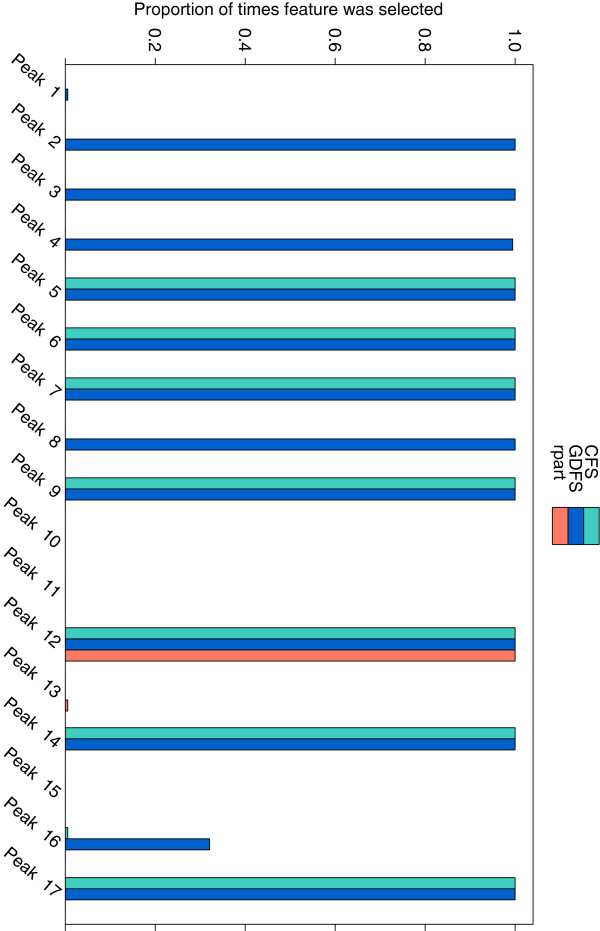
**Features selected from the lung cancer data.** The proportion of times, out of 184 cross-validation runs, that each glycan peak in the lung cancer dataset was selected by (**a**) the GDFS method (blue), (**b**) CFS (green), and (**c**) rpart (orange). Features were selected by leave-one-out cross-validation in each case.

As it is extremely difficult to distinguish between different stages of lung cancer, all 5 stages of cancer were combined for statistical analysis. Feature selection was carried out to identify a set of features (glycan peaks) that differ between the chromatograms of the control and lung cancer cases. Since all models were fitted using leave-one-out cross-validation, feature selection was carried out 184 times for each model, omitting a different observation each time. This means that the same set of features were not picked out in each cross-validation run. Figure 5 shows the proportion of times each feature (glycan peak) was selected by each feature selection method.

All three methods are quite consistent in the features they select. The GDFS method (blue) identifies 11 peaks, CFS (green) identifies 7 peaks, and rpart (orange) identifies only one peak in all class validation runs. All three methods select peak 12 as being an important feature for differentiating between control and lung cancer cases. Table 1 lists features that were selected at least 90% of the time for each method. Tabulated alongside are the predominant glycan structures associated with the selected glycan peaks. These were identified from Royle et al. [9] and verified by exoglycosidase digestions, as described by Arnold et al. [23].

**Table 1:** Feature selection for lung cancer data

	**GDFS**	**CFS**	**rpart**	**Predominant glycans(GDFS method)**
Peak 1	✗	✗	✗	
Peak 2	✓	✗	✗	A2
Peak 3	✓	✗	✗	FA2
Peak 4	✓	✗	✗	FA2B, A2[3]G1, A2[6]G1, M5
Peak 5	✓	✓	✗	FA2[3]G1, FA2[6]G1, FA2[3]BG1, FA2[6]BG1
Peak 6	✓	✓	✗	A2G2, A2BG2, A2[3]G1S1, A2[6]G1S1
Peak 7	✓	✓	✗	FA2G2, FA2BG2,FA2[3]G1S1, FA2[6]G1S1
Peak 8	✓	✗	✗	A2G2S1, A2BG2S1
Peak 9	✓	✓	✗	A3G3S2, A3BG3S2, A2F1G2S2
Peak 10	✗	✗	✗	
Peak 11	✗	✗	✗	
Peak 12	✓	✓	✓	A3G3S2, A3BG3S2, A2F1G2S2
Peak 13	✗	✗	✗	
Peak 14	✓	✓	✗	A3F1G3S3
Peak 15	✗	✗	✗	
Peak 16	✗	✗	✗	
Peak 17	✓	✓	✗	A4G4LacS4, A4F2G3S4

Features selected from the lung cancer dataset (control vs. cancer cases) by the proposed GDFS method (GDFS), correlation-based feature selection (CFS), and recursive partitioning (rpart). Features that were selected in 90% or more of the cross-validation models are marked with ✓. Also listed are the predominant glycan structures corresponding to each selected peak. Detailed *N*-glycan composition of human serum was described in [9] and these peaks were also assigned in [23]. Nomenclature has been used according to [9,50]: all *N*-glycans have two core GlcNAcs; F at the start of the abbreviation indicates a core fucose *α*1-6 to inner GlcNAc; Man (x), number (x) of mannose on core GlcNAcs; A(x), number(x) of antenna (GlcNAc) on trimannosyl core; B, bisecting GlcNAc linked *β*1-4 to *β*1-3 mannose; F(x), number (x) of fucose linked *α*1-3 to antenna GlcNAc, G(x), number (x) of galactose on antenna; [3]G1 and [6]G1 indicates that the galactose is on the antenna of the *α*1-3 or *α*1-6 mannose; S(x), number of sialic acids on antenna.

Structural assignments of *N*-glycans to the peaks on a HILIC chromatogram are made using the Glycobase software ( http://glycobase.nibrt.ie/glycobase/show_nibrt.action). Campbell et al. [51] provide further details.

**Figure 6:**
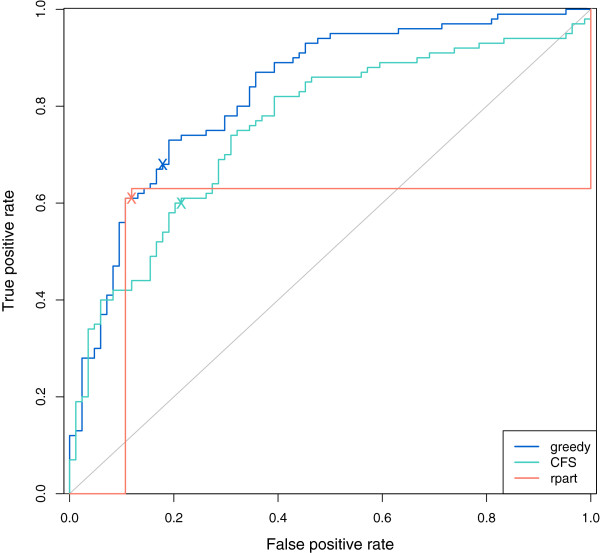
**ROC curve for lung cancer classification.** ROC curves for lung cancer classifications (control vs. cancer cases) were constructed for the proposed GDFS method (blue), CFS (green), and rpart (orange). In each case, the posterior probability of belonging to the lung cancer group was calculated for each observation j using features selected, and model parameters estimated, with observation j omitted (leave-one-out cross-validation). ROC curves were constructed from these posterior probabilities using the ROCR package in R [40]. ‘X’ marks the 0.5 classification threshold on each ROC curve.

Table 2 shows cross-tabulations of the true group membership with the classifications assigned by each feature selection method using a MAP classification rule. Table 3 lists measures of classification performance for the three methods. The GDFS method outperforms both CFS and rpart on all measures of classification performance, with a cross-validation error of 0.255, compared with 0.266 for rpart and 0.315 for CFS. It has a sensitivity rate that is 8% higher than the sensitivity rate for rpart and 9% higher than for CFS. Figure 6 shows ROC curves for the GDFS method (blue), the CFS (green), and rpart (orange). The 0.5 thresholds for each are marked “X” and correspond to classifications obtained from a MAP classification rule. The AUC for the GDFS method (0.83) is larger than for the other two methods (Table 3), reflecting its superior classification performance.

**Table 2:** Lung cancer data classifications

		**GDFS**		**CFS**		**rpart**	
		**Control**	**Cancer**	**Control**	**Cancer**	**Control**	**Cancer**
*True groups*	Control	69	15	66	18	74	10
	Cancer	32	68	40	60	39	61

Statistical classifications of the lung cancer dataset (control vs. cancer cases) from the proposed GDFS method (GDFS), correlation-based feature selection (CFS), and recursive partitioning (rpart). In each case, posterior group probabilities were calculated for each observation *j* using features selected, and model parameters estimated, with observation j omitted (leave-one-out cross-validation). Observations were then classified using a MAP classification rule. This table shows the cross-tabulations of true group membership with the assigned classifications from GDFS, CFS and rpart.

**Table 3:** Lung cancer data classification performance

	**Cross-validation error**	**Kappa**	**Sensitivity**	**AUC**
GDFS	0.255	0.493	0.680	0.830
CFS	0.315	0.378	0.600	0.757
rpart	0.266	0.478	0.610	0.562

Evaluation of classification performance from feature selection in the lung cancer dataset (control vs. cancer cases) for the proposed GDFS method (GDFS), correlation-based feature selection (CFS), and recursive partitioning (rpart). Reported are the cross-validation error (misclassification rate), Cohen’s kappa statistic, sensitivity, and AUC (corresponding to ROC curves in Figure 4) for the statistical classifications from each method.

### Prostate cancer data

Jemal et al. [48] observed that, globally, prostate cancer is the second most frequently diagnosed cancer in males and is the sixth most common cause of cancer death in males, based on figures from 2008. Prostate cancer is one of the most commonly diagnosed cancers in men. Prostate specific antigen (PSA) is a glycoprotein that is currently used as a clinical biomarker for this disease, but this glycoprotein is lacking in sensitivity and specificity. In fact, the U.S. Preventative Services Task Force (USPSTF) have recently issued a draft recommendation against PSA screening [52], after concluding that PSA-based screening for prostate cancer results in little or no reduction in the prostate cancer-specific mortaility. They also suggest that the screening may do more harm than good, due to the harms associated with evaluations or treatment carried out subsequent to screening. Several other potential biomarkers for this disease have been identified, but none that appear to be sensitive or specific enough for clinical use. Thus, there is an urgent need for further developments in this area.

Saldova et al. [24] conducted a study to investigate whether patterns of glycosylation are useful in differentiating between cases of prostate cancer and benign prostate hyperplasia (BPH). BPH is an enlargement of the prostate gland and is very common in men, especially as they age. BPH can present similar symptoms to prostate cancer and is also associated with elevated PSA levels. It would be extremely useful to identify a biomarker that can distinguish between these conditions. The study by Saldova et al. [24] was carried out using 34 prostate cancer cases (consisting of 17 cases with Gleason score 5 and 17 cases with Gleason score 7) and 13 men with BPH. The Gleason score is a currently used measure of disease severity. It ranges from 2 to 10, with a higher score indicating a more advanced stage of disease.

#### Variable selection for prostate cancer data - cancer vs. BPH

**Figure 7:**
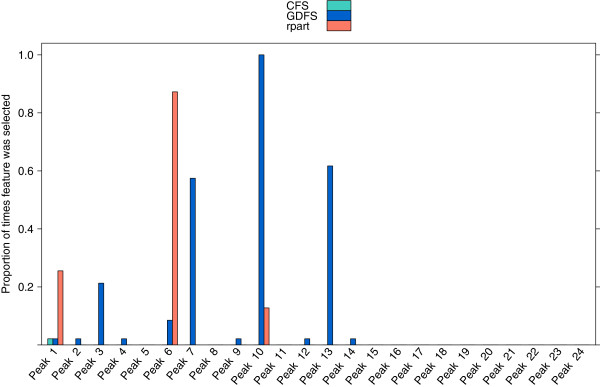
**Features selected from the prostate cancer data (prostate cancer vs. BPH).** The proportion of times, out of 47 cross-validation runs, that each glycan peak in the prostate cancer dataset (BPH vs. prostate cancer) was selected by (**a**) the proposed GDFS method (blue), (**b**) CFS (green), and (**c**) rpart (orange). Features were selected by leave-one-out cross-validation in each case.

Feature selection was performed to select a set of features (glycan peaks) that differ between the chromatograms of the 34 prostate cancer cases and the 13 BPH cases in the prostate cancer dataset. Three methods are compared here; the proposed GDFS method, correlation-based feature selection (CFS), and recursive partitioning (rpart). Since the models were fitted using leave-one-out cross-validation, feature selection was carried out 47 times in each case. Figure 7 shows the proportion of times each feature (glycan peak) was selected out of the 47 cross-validation runs, for each feature selection method. Correlation-based feature selection consistently selects no features for this dataset. This is due to the fact that when each of the compositional components are discretized according to the method of Fayyad and Irani [38], all features are assigned to be single level factors. Where this is the case, the “merit” of any selected feature set is equal to zero, and hence an empty feature set is returned. Table 4 lists the peaks that were identified 90% of the time or more for each method. The GDFS method most commonly selected peaks 10 (less frequently) and 13. The rpart method chose peak 6 most frequently, but for less than 90% of the cross-validation runs. Table 4 also lists the predominant glycan structures corresponding to these most commonly selected glycan peaks.

**Table 4:** Variable selection for prostate cancer data (prostate cancer vs. BPH)

	**GDFS**	**CFS**	**rpart**	**Predominant glycans (GDFS method)**
Peak 1	✗	✗	✗	
Peak 2	✗	✗	✗	
Peak 3	✗	✗	✗	
Peak 4	✗	✗	✗	
Peak 5	✗	✗	✗	
Peak 6*	✗	✗	✗	FA2[3]G1, FA2[6]BG1
Peak 7	✗	✗	✗	
Peak 8	✗	✗	✗	
Peak 9	✗	✗	✗	
Peak 10	✓	✗	✗	FA2G2, FA2[6]G1S1, FA2[6]BG1S1
Peak 11	✗	✗	✗	
Peak 12	✗	✗	✗	
Peak 13**	✗	✗	✗	A2BG2S1
Peaks 14 - 24	✗	✗	✗	

Features selected from the prostate cancer dataset (prostate cancer vs. BPH cases) by the proposed GDFS method (GDFS), correlation-based feature selection (CFS), and recursive partitioning (rpart). Features that were selected in 90% more of the cross-validation models are marked with ✓. Also listed are the predominant glycan structures corresponding to each selected peak. Detailed *N*-glycan composition of human serum was described in Royle et al. [9], and peak 10 was also assigned in Saldova et al. [24]. *Peak 6 was the most commonly identified feature by the rpart method, although it was selected less than 90% of the time. **Peak 13 was selected more than 60% of the time by the GDFS method.

Table 5 shows cross-tabulations between the true group membership and the classifications assigned by each feature selection method using MAP classifications. No classifications were obtained for correlation-based feature selection, since most of the cross-validation models returned no features.

**Table 5:** Prostate cancer data classifications (prostate cancer vs. BPH)

		**GDFS**		**CFS**		**rpart**	
		**BPH**	**Cancer**	**BPH**	**Cancer**	**BPH**	**Cancer**
*True groups*	BPH	1	12	-	-	7	6
	Cancer	13	21	-	-	20	14

Statistical classifications of the prostate cancer dataset (prostate cancer vs. BPH cases) from the proposed GDFS method (GDFS), correlation-based feature selection (CFS), and recursive partitioning (rpart). In each case, posterior group probabilities were calculated for each observation *j* using features selected, and model parameters estimated, with observation j omitted (leave-one-out cross-validation). Observations were then classified using a MAP classification rule. This table shows the cross-tabulations of true group membership with the assigned classifications from the the GDFS, CFS, and rpart methods.

Table 6 compares the feature selection methods on four different measures of classification performance. Neither the GDFS search or the rpart method classify the data well, but the GDFS method outperforms rpart at a 0.5 probability cut-off, having a slightly lower cross-validation error rate and a higher sensitivity rate.

**Table 6:** Prostate cancer data classification performance (prostate cancer vs. BPH)

	**Cross-validation error**	**Kappa**	**Sensitivity**	**AUC**
GDFS	0.532	-0.298	0.618	0.274
rpart	0.553	-0.037	0.412	0.371

**Figure 8:**
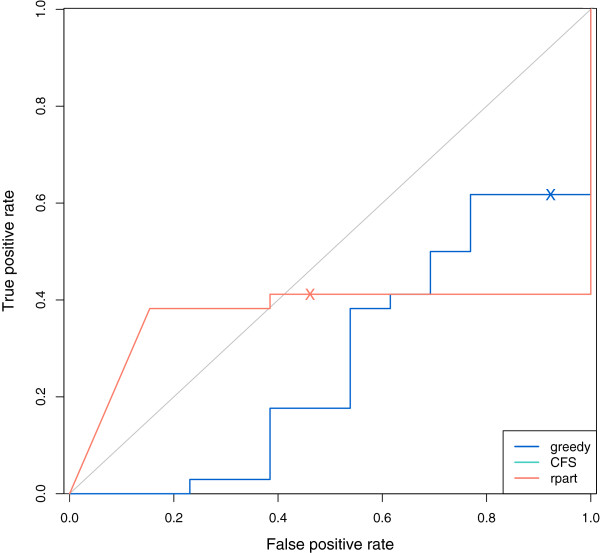
**ROC curve for prostate cancer classification (prostate cancer vs. BPH).** ROC curves for prostate cancer classifcations (prostate cancer vs. BPH cases) were constructed for the proposed GDFS method (blue) and rpart (orange). The results for CFS are not included here, as the method selected no features in all but one of the cross-validation runs. In each case, the posterior probability of belonging to the prostate cancer group was calculated for each observation j using features selected, and model parameters estimated, with observation j omitted (leave-one-out cross-validation). ROC curves were constructed from these posterior probabilities using the ROCR package in R [40]. ‘X’ marks the 0.5 classification threshold on each ROC curve.

Evaluation of classification performance from feature selection in the prostate cancer dataset (prostate cancer vs. BPH cases) for the proposed GDFS method (GDFS), correlation-based feature selection (CFS), and recursive partitioning (rpart). Reported are the cross-validation error (misclassification rate), Cohen’s kappa statistic, sensitivity, and AUC (corresponding to ROC curves in Figure 8) for the statistical classifications from each method.

Figure 8 shows ROC curves for the GDFS method (blue) and rpart (orange). The 0.5 probability threshold is marked “X” on each and correspond to classifications obtained from a MAP classification rule. The ROC curve for the rpart method has a higher AUC value (0.371) than the AUC value for the GDFS method (0.274). Again, both methods perform poorly, suggesting that there is little difference between BPH and cancer groups for this dataset.

#### Variable selection for prostate cancer data - disease progression

In addition to the separation of BPH from prostate cancer samples, it is desirable to see whether the serum *N*-glycan profile changes as prostate cancer progresses. Gleason scores are assigned to prostate cancer cases based on the microscopic appearance of the cancerous tissue. They range from 2 to 10, with grade 10 having the worst prognosis. Feature selection was carried out on the prostate cancer samples from the study by Saldova et al. [24], to investigate whether there differences in the chromatograms of the 17 Gleason 5 and 17 Gleason 7 cases. Three feature selection methods are compared; the proposed GDFS method, correlation-based feature selection (CFS), and a recursive partitioning (rpart).

**Figure 9:**
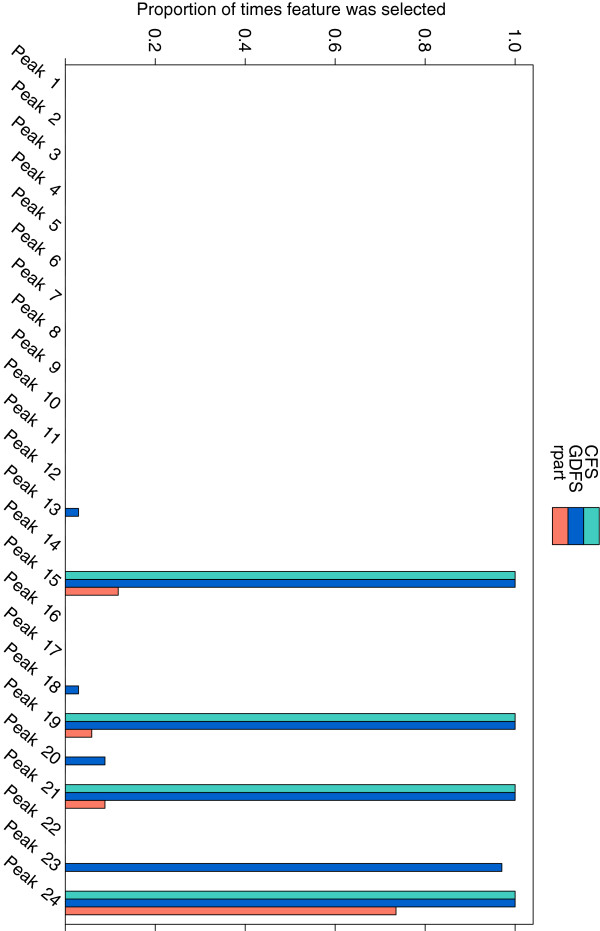
**Features selected from the prostate cancer data (Gleason 5 vs. Gleason 7).** The proportion of times, out of 34 cross-validation runs, that each glycan peak in the lung cancer dataset was selected by (**a**) the proposed GDFS method (blue), (**b**) CFS (green), and (**c**) rpart (orange). Features were selected by leave-one-out cross-validation in each case.

Since all models were fitted using leave-one-out cross-validation, feature selection was carried out 34 times for each method, omitting a different observation each time. Figure 9 shows the proportion of times each feature (glycan peak) was selected for the three feature selection methods. The GDFS search and CFS methods were very consistent in the features selected over the cross-validation runs, while the rpart method was somewhat less consistent. Table 7 marks the glycan peaks that were selected in 90% or more of the cross-validation runs. Also tabulated are the predominant glycans that correspond to these selected peaks. The GDFS method consistently selects 5 peaks, and CFS frequently selects the same peaks, with the exception of peak 24. The rpart classification tree method is not very consistent and does not select any peak more than 80% of the time.

**Table 7:** Variable selection for prostate cancer data (Gleason 5 vs. Gleason 7)

	**GDFS**	**CFS**	**rpart**	**Predominant glycans (GDFS method)**
Peaks 1–14	✗	✗	✗	
Peak 15	✓	✓	✗	FA2BG2S1, A3G3
Peak 16	✗	✗	✗	
Peak 17	✗	✗	✗	
Peak 18	✗	✗	✗	
Peak 19	✓	✓	✗	A3G3S2
Peak 20	✗	✗	✗	
Peak 21	✓	✓	✗	A3G3S3
Peak 22	✗	✗	✗	
Peak 23	✓	✗	✗	A4G4S4
Peak 24*	✓	✓	✗	A4F1G4S4

Features selected from the prostate cancer dataset (Gleason 5 vs. Gleason 7 cases) by the proposed GDFS method (GDFS), correlation-based feature selection (CFS), and recursive partitioning (rpart). Features that were selected in 90% more of the cross-validation models are marked with ✓. Also listed are the predominant glycan structures corresponding to each selected peak. Detailed *N*-glycan composition of human serum was described in Royle et al. [9], and these peaks were also assigned in Saldova et al. [24]. *Peak 24 was the one most frequently selected by rpart, but less than 80% of the time.

**Figure 10:**
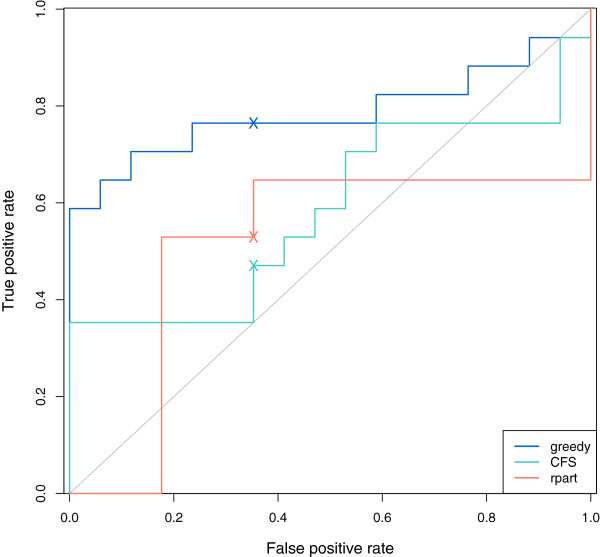
**ROC curve for prostate cancer classification (Gleason 5 vs. Gleason 7).** ROC curves for prostate cancer classifcations (Gleason 5 vs. Gleason 7 cases) were constructed for the proposed GDFS method (blue), CFS (green), and rpart (orange). In each case, the posterior probability of belonging to the Gleason 7 group was calculated for each observation j using features selected, and model parameters estimated, with observation j omitted (leave-one-out cross-validation). ROC curves were constructed from these posterior probabilities using the ROCR package in R [40]. ‘X’ marks the 0.5 classification threshold on each ROC curve.

Table 8 shows cross-tabulations between the true group membership and the classifications assigned by each feature selection method (using a MAP classification rule), while Table 9 compares the methods on four different measures of classification performance. The GDFS search gives the smallest cross-validation error of 0.294. It also has higher sensitivity for detecting more severe cases of prostate cancer (Gleason 7). Figure 10 shows ROC curves for the GDFS method (blue), CFS (green), and rpart (orange). The 0.5 thresholds for each are marked “X” and correspond to classifications assigned by a MAP classification rule. From the ROC curves, it is clear that the GDFS method markedly outperforms the other two methods, with a larger AUC of 0.785 (Table 9).

**Table 8:** Prostate cancer data classifications (Gleason 5 vs. Gleason 7)

		**GDFS**		**CFS**		**rpart**	
		**Gleason 5**	**Gleason 7**	**Gleason 5**	**Gleason 7**	**Gleason 5**	**Gleason 7**
*True groups*	Gleason 5	11	6	11	6	11	6
	Gleason 7	4	13	9	8	8	9

Statistical classifications of the prostate cancer dataset (Gleason 5 vs. Gleason 7 cases) from the proposed GDFS method (GDFS), correlation-based feature selection (CFS), and recursive partitioning (rpart). In each case, posterior group probabilities were calculated for each observation *j* using features selected, and model parameters estimated, with observation j omitted (leave-one-out cross-validation). Observations were then classified using a MAP classification rule. This table shows the cross-tabulations of true group membership with the assigned classifications from the GDFS, CFS and rpart methods.

**Table 9:** Prostate cancer data classification performance (Gleason 5 vs. Gleason 7)

	**Cross-validation error**	**Kappa**	**Sensitivity**	**AUC**
GDFS	0.294	0.412	0.765	0.785
CFS	0.441	0.118	0.471	0.585
rpart	0.412	0.176	0.529	0.512

Evaluation of classification performance from feature selection in the prostate cancer dataset (Gleason 5 vs. Gleason 7 cases) for the proposed GDFS method (GDFS), correlation-based feature selection (CFS), and recursive partitioning (rpart). Reported are the cross-validation error (misclassification rate), Cohen’s kappa statistic, sensitivity, and AUC (corresponding to ROC curves in Figure 10) for the statistical classifications from each method.

### Search strategy and computational complexity

For a dataset of dimension *p*, the cardinality of the feature space increases exponentially with *p*. An exhaustive search over this space would involve an evaluation of all possible solutions and for this problem has complexity 2^*p*^. That is, for *p* variables, there are 2^*p*^ possible solutions to the feature selection problem. An exhaustive search would certainly be possible for a relatively small number of variables, but the computational complexity increases quickly. A dataset with 24 variables has 16,777,216 possible solutions in the feature selection problem!

Glycan chromatography data being produced is of a relatively low dimensionality at present. It has been found that there are 117 glycans in human serum [9], and therefore, it can be expected that the number of variables in the glycan chromatography data will increase as technology becomes more advanced. For example, Bones et al. [10] recently showed that ultra performance liquid chromatography (UPLC) allows for the quantification of the glycan pool by a chromatogram consisting of 53 glycan peaks, under certain experimental conditions.

Table 10 shows the expected behaviour of the GDFS algorithm with increasing data dimensionality (from *p*=20 to *p*=100), using datasets simulated from Dirichlet distributions. The parameters for a subset of the compositional variables (approx. a third) were set to differ across two groups of 100 observations each. Reported are the run times (in seconds) for the GDFS, correlation-based feature selection, and recursive partitioning methods. The GDFS and correlation-based feature selection methods were implemented manually in R, while recursive partitioning was implemented using the rpart package in R [41]. Alongside the run times are the discrepancies between the true and selected feature sets, calculated as the sum of the number of incorrectly selected features and the number of true features that were not selected. From this table, it is clear that while the GDFS algorithm is less efficient computationally, it has a much higher accuracy than the other two methods for our simulated datasets.

**Table 10:** Computational efficiency of the GDFS method compared with CFS and rpart

***p***	**GDFS**		**CFS**		**rpart**	
10	3.29	(0)	2.065	(0)	0.02	(2)
20	52.246	(0)	11.527	(2)	0.023	(6)
30	113.702	(0)	23.395	(5)	0.029	(9)
40	249.751	(2)	30.866	(8)	0.038	(12)
50	498.445	(1)	83.885	(10)	0.043	(16)
60	609.841	(0)	415.525	(4)	0.05	(19)
70	962.695	(2)	828.434	(3)	0.083	(22)
80	1902.347	(0)	696.083	(10)	0.068	(26)
90	1516.234	(1)	1286.167	(9)	0.078	(28)
100	2059.3	(1)	812.16	(17)	0.096	(31)

Time taken (in seconds) to carry out feature selection using a greedy search approach for simulated datasets of increasing data dimensionality, p. Tabulated alongside are the run times for CFS, and rpart corresponding to these same datasets. Data were simulated from ordinary Dirichlet distributions across two groups, with 100 observations in each group. Approximately one third of the variables were set to differ between groups (“grouping variables”). Reported in brackets beside the run times are the number of discrepancies between the true set of grouping variables and the selected feature set. The number of discrepancies was calculated as the sum of the number of variables that were incorrectly selected as features and the number of true grouping variables that were not selected by the selection algorithm.

Murphy, Dean, and Raftery [22] used the headlong search strategy proposed by Badsberg [20] in their variable selection. They add (or remove) the first variable whose BIC difference is greater than (or less than) a pre-specified value. This removes the necessity to search through all variables at each iteration, reducing computational time dramatically over an ordinary greedy search strategy. However, they state that the variables selected using this method may change depending on the initial ordering of variables in the dataset. They preferred this approach, as they had over 1000 variables to consider in their application. Since glycan chromatography datasets are relatively low-dimensional, we avoid this issue by using considering all possibilities of variables to add or remove at each iteration.

## Conclusions

Biomarker discovery is of the utmost importance for disease discovery and treatment. The field of glycobiology shows great potential in this area and is continually improving technologies to advance research into the identification and validation of glycan biomarkers. Glycan analysis is commonly carried out using high-throughput chromatography techniques, that give rise to compositional data. The compositional nature of the data is commonly ignored in statistical analysis, mainly due to lack of awareness of the special considerations that are required for the analysis of such data.

There is a substantial need in the field of glycobiology for a statistical toolbox of suitable methods for dealing with the compositional glycan chromatography data. This article hopes to contribute a novel method for feature selection that could be used for identifying sets of potential biomarkers. The method carries out a greedy search over the space of all possible sets of features, seeking the set of features that best discriminates between a set of defined groups in the data. The generalized Dirichlet distribution and its marginal, the beta distribution, are used to model compositional components (variables), since they suitable for proportional data. The BIC is used for model selection.

This methodology was tested on two glycan chromatography datasets, from the lung cancer study by Arnold; et al. [23] and the prostate cancer study by Saldova et al. [24]. Two other well-established methods were applied to these datasets for comparison - correlation based feature selection (CFS) and a recursive partitioning method for classification tree construction (rpart package in R [40]). For the lung cancer dataset, a set of 11 peaks are consistently identified by the GDFS method as differing between the lung cancer and clinical control cases (Table 1). Peaks 12, 14, and 17, included in this selected feature set, contain the sialyl Lewis x (SLe ^*X*^) epitope, which is known to be increased in cancer and important for cancer progression [25]. For the prostate cancer dataset, peaks 10 and 13 are consistently identified by the GDFS method as potential glycan biomarkers for differentiating between BPH and prostate cancer. peak 10 contains core-fucosylated bi-antennary glycans, and peaks 10 and 13 contain bisected bi-antennary glycans. Our findings are consistent with previous results showing that core-fucosylation is altered in cancer and bisects are decreased in cancer [53]. Regarding separation of different disease stages, five *N*-glycan peaks were selected by the GDFS method (peaks 15, 19, 21, 23, and 24) as differing between Gleason 5 and Gleason 7 cases. This indicates a decrease in triantennary trigalactosylated glycans and in tetraantennary tetrasialylated outer arm fucosylated glycans and an increase in tetraantennary tetrasialylated glycans in Gleason 7 compared with Gleason 5 prostate cancer patients [24].

In general, the proposed GDFS method outperformed both CFS and rpart on classification performance, although it is somewhat slower computationally. Importantly, the sensitivity of the classifiers was largest for the GDFS method in all cases, meaning that more of the actual lung cancer cases were detected. From our results, we conclude that the proposed GDFS method provides a useful tool for feature selection in compositional glycan chromatography data.

This method has been developed specifically with glycan chromatography data in mind and accounts for the special constraints on a compositional dataset, since the data are modelled in a simplex sample space. It has been used for feature selection in the context of supervised learning, where the data have a known group structure, but may easily be extended for use with unsupervised learning methods, such as model-based clustering, as in Raftery and Dean [19].

## Appendix

### A. change of variable rule

Let *Y* be a continuous random variable with probability density function *f*_*y*_(*y*), and let $\tilde{y}=g(y)$ be an invertible function of Y, with inverse function $\tilde{y}=h(y)$. Then the probability density function of *Y* may be written in terms of the probability density function of $\tilde{Y}$ as:

(37) $$\begin{array}{ll} f_{y}(y)\;\; & =f_{\;\;\tilde{y}}(h(y))|h^{\prime }(y)|\; \end{array}$$

where *h*^*′*^(*y*) is the derivative of $\tilde{y}=h(y)$ with respect to *y*.

### B. Derivation of the generalized Dirichlet probability density function

If **Y**=(*Y*_1_,*Y*_2_,…,*Y*_*p*_) is a unit-sum composition following a generalized Dirichlet distribution, then $\tilde{Y}$ is a completely neutral vector, meaning that the components of the vector

(38) $$\begin{array}{ll} \tilde{Y}\;\; & =\;\;h(Y)=\left(\frac{Y_{1}}{1-S_{0}},\frac{Y_{2}}{1-S_{1}},\ldots ,\frac{Y_{p}}{1-S_{p-1}}\right)\; \end{array}$$

are mutually independent, where *S*_0_=1 and $S_{j}=\overset{j}{\underset{m=1}{\sum }}S_{m}$ for *m*=1,2,…,*p*. Note that the last component of $\tilde{Y}$ is degenerate, since it is equal to one. Since **Y** is a generalized Dirichlet random vector, the marginal distributions of the elements of $\tilde{Y}$ are beta distributions, so that ${\tilde{Y}}_{j}∼\text{beta}(\alpha_{j},\beta_{j})$ for *j*=1,2,…,*p*−1.

Denote the probability density function of *Y*_*j*_, conditional on (*Y*_1_,*Y*_2_,…,*Y*_*j*−1_), by *f*_*j*_ for *j*=1,2,…,*p*−1. The probability density function for $\tilde{Y}$ is the product of *p*−1 independent beta distributions. Thus, the probability density function for **Y** may easily be derived in terms of the probability density function for $\tilde{Y}$. Firstly, the density function for **Y** can be written as the product of *p*−1 conditional distributions

(39) $$\begin{array}{lll} f(y_{i}) & =f\left(y_{i1},y_{i2},\ldots ,y_{i(p-1)}\right)\; & \\ & =f_{1}(y_{i1})f_{2}(y_{i2}|y_{i1})\ldots \; & \\ & \;f_{p-1}(y_{i(p-1)}|y_{i1},y_{i2},\ldots ,y_{i(p-2)}),\; \end{array}$$

using the rules of conditional probability and because one component of a compositional vector is degenerate, conveniently chosen here to be *Y*_*p*_. Making the change of variable ${\tilde{y}}_{\mathrm{ij}}=h(y_{\mathrm{ij}})=y_{\mathrm{ij}}/(1-s_{i,j-1})$ for *j*=1,2,…,*p*−1, gives rise to Jacobian terms

(40) $$\begin{array}{ll} \frac{\partial }{\partial y_{\mathrm{ij}}}\left\{\frac{y_{\mathrm{ij}}}{1-s_{i,j-1}}\right\}\;\; & =\;\;\frac{1}{1-s_{i,j-1}}\; \end{array}$$

for *j*=1,2,…,*p*−1. Denoting the probability density function for each ${\tilde{Y}}_{j}$ by *g*_*j*_, and noting that ${\tilde{Y}}_{j}∼\text{beta}(\alpha_{j},\beta_{j})$, distributed independently of (*Y*_1_,*Y*_2_,…,*Y*_*j*−1_) gives

$$\begin{array}{lll} f(y_{i}) & =\;\;g_{1}\left(\frac{y_{i1}}{1-s_{i,0}}\right)g_{2}\left(\frac{y_{i2}}{1-s_{i,j-1}}\left|\right)y_{i1}\right)\ldots \; & \\ & \;\;g_{p-1}\left(\frac{y_{i(p-1)}}{1-s_{i,p-2}}\left|\right)y_{i1},y_{i2},\ldots ,y_{i(p-2)}\right)\; & \\ & \;\times \left(\frac{1}{1-s_{i,0}}\right)\left(\frac{1}{1-s_{i,1}}\right)\ldots \left(\frac{1}{1-s_{i,p-1}}\right)\; & \\ & \;\;\;\;\;\;\;\;\text{. . . Jacobian terms}\; & \\ & =\;\overset{p-1}{\underset{j=1}{\prod }}g_{j}\left(\frac{y_{\mathrm{ij}}}{1-s_{i,j-1}}\right)\left(\frac{1}{1-s_{i,j-1}}\right)\;\\ & =\overset{p-1}{\underset{j=1}{\prod }}\frac{1}{B(\alpha_{j},\beta_{j})}{\left(\frac{y_{\mathrm{ij}}}{1-s_{i,j-1}}\right)}^{\alpha_{j}-1}\; & \\ & \;\times {\left(1-\frac{y_{\mathrm{ij}}}{1-s_{i,j-1}}\right)}^{\beta_{j}-1}{\left(1-s_{i,j-1}\right)}^{-1}\; & \\ & =\;\overset{p-1}{\underset{j=1}{\prod }}\;\;\frac{1}{B(\alpha_{j},\beta_{j})}y_{\mathrm{ij}}^{\alpha_{j}-1}{\left(\;1\;-\;s_{i,j-1}\right)}^{\;1-\alpha_{j}-\beta_{j}}\left(\;1\;-s_{i,j-1}\;-y_{\mathrm{ij}}\right){\;}^{\beta_{j}-1}\; & \\ & =\;\overset{p-1}{\underset{j=1}{\prod }}\frac{1}{B(\alpha_{j},\beta_{j})}\;\;y_{\mathrm{ij}}^{\alpha_{j}-1}{\left(1-s_{i,j-1}\right)}^{1-\alpha_{j}-\beta_{j}}{\left(1-s_{i,j}\right)}^{\beta_{j}-1}\; & \\ & =\;\left[\overset{p-1}{\underset{j=1}{\prod }}\frac{1}{B(\alpha_{j},\beta_{j})}\;\;y_{\mathrm{ij}}^{\alpha_{j}-1}\right]\;\left[\overset{p-1}{\underset{j=1}{\prod }}{\left(1-s_{i,j-1}\right)}^{\beta_{j-1}-\alpha_{j}-\beta_{j}}\right]\; & \\ & \;\times {\left(1-s_{i,j-1}\right)}^{\beta_{p-1}-1}\; & \end{array}$$

since 1−*s*_*i*,0_=1. Note that 1−*s*_*i*,*p*−1_=*y*_*ip*_, and then the probability density function for **Y**∼GD(*α*_1_,*β*_1_,…,*α*_*p*−1_,*β*_*p*−1_) is

$$\begin{array}{lll} f(y_{i})\; & =\;y_{\mathrm{ip}}^{\beta_{p-1}-1}\overset{p-1}{\underset{j=1}{\prod }}\frac{1}{B(\alpha_{j},\beta_{j})}y_{\mathrm{ij}}^{\alpha_{j}-1}\; & \\ & \;\times {\left(1-s_{i,j-1}\right)}^{\beta_{j-1}-\alpha_{j}-\beta_{j}}\;\;\; & \end{array}$$

### C. Construction of classification trees using recursive partitioning

Briefly, the classification trees fitted here are constructed by splitting observations into subsets that give the best separation between the set of known groups in the data. Subsets of observations are represented by nodes in the classification tree. Each node is labelled by the predominant class of observations at that node, and the misclassification error for any node is then the proportion of observations at that node that don’t belong to the predominant class.

All observations are included in the root node. A binary split of the observations at a given node is made by selecting the feature (variable), and the split threshold for that feature that give the best separation of the classes. Here, the feature set and cut threshold are selected to minimize the Gini index, a measure of “impurity”, or the average misclassification error for the child nodes resulting from a binary split. Then the observations at this node are split into two child nodes according to whether their observed values of the selected feature lie above or below the split threshold. The process is then repeated for the resulting child nodes.

The tree is expanded recursively in this manner until some stopping criterion is met, for example, until only observations in the same class are present at leaf nodes. To avoid over-fitting, the tree is then pruned back by snipping off nodes from the bottom up, selecting the branches to be pruned using a cost complexity measure:

(41) $$\;\begin{array}{cc} \;\;\;\;R_{\alpha } & =\text{Misclassification Error}+(\alpha \times \text{Number of splits}) \end{array}$$

where *α* is a penalty term that controls the size of the tree. The final tree is then chosen as the “pruned” version of the full tree, that minimizes the cost complexity, *R*_*α*_. The value of *α* is estimated using the “1-SE” rule proposed by Brieman et al. [27]. If *α*∈[0,*∞*], then this interval may be partitioned into a set of sub-intervals (*I*_1_,*I*_2_,…,*I*_*k*_), such that any value of *α* in the interval *I*_*j*_ will give rise to the same subtree obtained from pruning the expanded tree by minimizing *R*_*α*_. The **rpart** function provides the cross-validated risk (average *R*_*α*_ value from ten-fold cross-validation) along with its standard error, evaluated at the range of complexity parameters *α* equal to geometric means of the maximum and minimum values for each interval (*I*_1_,*I*_2_,…,*I*_*k*_). Any cost complexity score within one standard error of the minimum is then marked as being equivalent to the minimum. The optimum value of the complexity parameter *α* is the one the gives is the simplest of set of models at the “minimum” cost complexity (or in other words, the largest value of *α*, since *α* is a penalty for complexity).

### D. Pseudo-code for correlation-based feature selection

Pseudocode for carrying out correlation-based feature selection for a set of variables (*X*1,*X*2,…,*XP*).

## Abbreviations

HILIC: Hydrophilic interaction liquid chromatography; CFS: Correlation-based feature selection; rpart: recursive partitioning method for the construction of classification trees; GDFS: Generalized Dirichlet feature selection; BIC: Bayesian information criterion

## Competing interests

The authors declare that they have no competing interests.

## Authors’ contributions

MG performed the statistical analysis and drafted the manuscript. RS carried out the experimental analysis and assisted with the drafting of the manuscript. MPC assisted with the interpretation of the data and critically revised the manuscript. PMR conceived of the scientific studies and participated in their design and coordination. TBM conceived of the statistical methodology and helped to critically revise the manuscript. All authors read and approved the final manuscript.
